# Metabolic Activation versus Masked Prodrugs: Bisubstrate Mimic Inhibitors of CoaBC’s PPCS Activity in Mycobacterium tuberculosis and Staphylococcus aureus

**DOI:** 10.1021/acsinfecdis.5c00047

**Published:** 2025-06-03

**Authors:** Timothy J. Kotzé, Konrad J. Mostert, Riyad Domingo, Xu Wang, Wessel J. A. Moolman, Hailey S. Butman, Abigail Pepin, Kyle T. McKay, Deon P. Neveling, Joanna C. Evans, Valerie Mizrahi, Willem A. L. van Otterlo, Cynthia S. Dowd, Erick Strauss

**Affiliations:** † Department of Biochemistry, 26697 Stellenbosch University, Stellenbosch 7600, South Africa; ‡ Department of Chemistry, 8367 The George Washington University, Washington DC 20052, United States; § Molecular Mycobacteriology Research Unit, Institute of Infectious Disease and Molecular Medicine and Department of Pathology, Faculty of Health Sciences, 63726 University of Cape Town, Observatory 7925, South Africa; ∥ Department of Chemistry & Polymer Science, Stellenbosch University, Stellenbosch 7600, South Africa

**Keywords:** bisubstrate mimics, metabolic activation, prodrug, coenzyme A, antimicrobials, PPCS

## Abstract

The bifunctional bacterial CoaBC is a coenzyme A (CoA) biosynthetic protein that serves as a validated bactericidal target in Mycobacterium tuberculosis (Mtb). In Staphylococcus aureus, it is the target of the natural product antibiotic CJ-15,801, which inhibits its phosphopantothenoylcysteine synthetase (PPCS) activity by forming a bisubstrate mimic of its reactive reaction intermediate *in situ* after metabolic activation by pantothenate kinase, the first CoA biosynthetic enzyme. We prepared PPCS bisubstrate mimics with various stable linkers that would also require metabolic activation and used purified Mtb and *S. aureus* enzymes to evaluate their inhibition. Additionally, we prepared masked prodrug versions of the phosphorylated (activated) form of CJ-15,801 and tested these and the bisubstrate mimics, as whole-cell inhibitors of Mtb and *S. aureus*. We demonstrate that such inhibitors hold promise for the development of antimicrobials targeting these organisms, although further structure–activity relationship studies are necessary to address current challenges and improve their potency.

## Graphical abstract

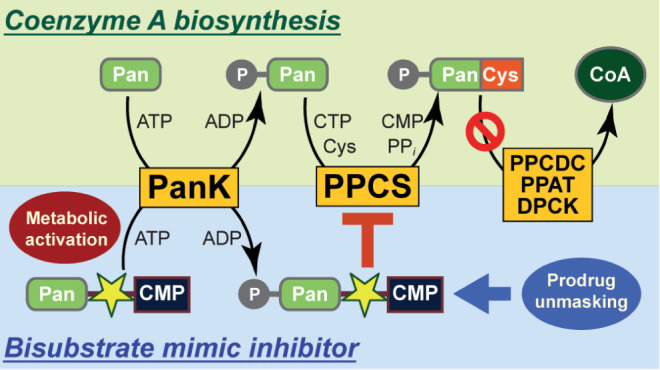

The rising threat of antimicrobial resistance (AMR)^1^ in pathogens of global concern—including Staphylococcus aureus and Mycobacterium tuberculosis (Mtb), the causative agent of tuberculosis (TB)—provides impetus for the discovery of novel antimicrobial drug candidates, preferably ones with modes of action that do not overlap with those of antimicrobial agents currently in clinical use. Targeting the biosynthesis of coenzyme A (CoA, **1**) offers promise in this context because of CoA’s essential role in central energy and fatty acid metabolism and because differences in how the pathway is constituted in humans and bacteria offers scope for selectivity. Consequently, several CoA biosynthetic enzymes have been targeted for inhibitor discovery in previous studies.^2–5^ Moreover, since persistent (nonreplicating but metabolically active) Mtb and *S. aureus* rely on several biochemical pathways that are CoA-dependent, the successful inhibition of CoA biosynthesis may also offer options for clearing chronic infections attributed to bacteria in this state.^6–9^

CoA is biosynthesized from pantothenic acid (Pan, vitamin B_5_, **2**) in five enzymatic steps following the *de novo* pathway, or by using pantetheine (PantSH, **3**) in the so-called salvage pathway, bypassing the phosphopantothenoylcysteine synthetase (PPCS) and phosphopantothenoylcysteine decarboxylase (PPCDC) catalyzed steps. In bacteria, these two activities are found on the bifunctional CoaBC protein (Figure 1A).^10,11^ However, the ubiquitous presence of Vanin pantetheinases that degrade PantSH to Pan and cysteamine in the human host renders the salvage pathway an unviable option for CoA biosynthesis at the host–pathogen interface.^12,13^ This is underscored by the genetic validation of CoaBC as a vulnerable target in Mtb *in vivo*, being required for Mtb’s growth and persistence in a mouse model, and with evidence that CoaBC depletion is bactericidal.^14^ In *S. aureus*, it was found that the natural product CJ-15,801 (**4**) selectively inhibits the PPCS activity of its CoaBC (*Sa*CoaBC) following activation by pantothenate kinase (PanK), the first enzyme in the pathway (Figure 1B).^15^ These findings, and several differences between human and bacterial enzymes—such as the distinction between monofunctional human PPCS and PPCDC enzymes and the bifunctional CoaBC in bacteria, and the preference of human PPCS to use ATP for activation, while the bacterial CoaBC’s PPCS activity requires CTP^10,16^ —have made CoaBC an attractive target for antimicrobial drug discovery.

**Figure 1:**
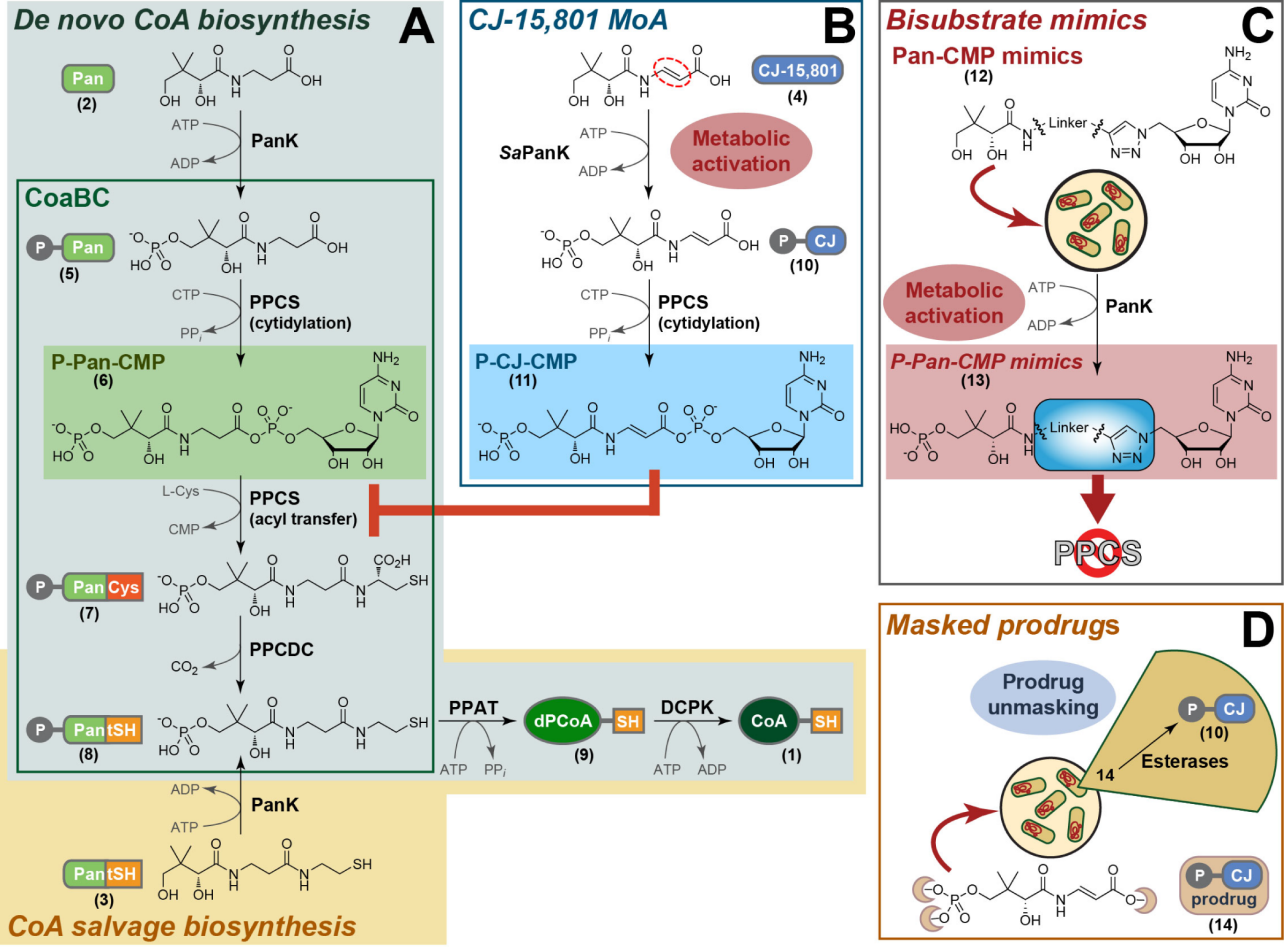
Coenzyme A (CoA) biosynthesis and strategies for the inhibition of its third step, phosphopantothenoylcysteine synthetase (PPCS). **A)** *De novo* CoA biosynthesis (boxed in gray) starts with the conversion of pantothenic acid (Pan, **2**) by pantothenate kinase (PanK) to 4′-phosphopantothenic acid (P-Pan, **5**). P-Pan is converted by PPCS to 4′-phosphopantothenoylcysteine (P-PanCys, **7**) by first transferring a cytidyl group to form the reactive 4′-phosphopantothenoyl-cytidylate (P-Pan-CMP, **6**) that acts as an acyl donor in reaction with l-cysteine. Phosphopantothenoylcysteine decarboxylase (PPCDC) then decarboxylates **7** to form 4′-phosphopantetheine (P-PantSH, **8**). In bacteria, the PPCS and PPCDC activities are colocated on the bifunctional CoaBC protein (framed in green). In the last two steps phosphopantetheine adenylyltransferase (PPAT) transfers an AMP group to **8** to give 3′-dephosphocoenzyme A (dPCoA, **9**), which is then phosphorylated by dephospho-coenzyme A kinase (DPCK) to give CoA (**1**). In organisms with promiscuous PanK enzymes that accept pantetheine (PantSH, **3**) as substrate to form **8** directly, CoA salvage biosynthesis (boxed in gold) can take place, bypassing CoaBC (PPCS and PPCDC). **B)** The natural product antistaphylococcal agent CJ-15,801 (**4**) acts by being metabolically activated through phosphorylation by *S. aureus* PanK (*Sa*PanK) to form 4′-phospho-CJ-15,801 (P-CJ, **10**). Cytidylylation by PPCS forms 4′-phospho-CJ-15,801-cytidylate (P-CJ-CMP, **11**), an unreactive bisubstrate mimic of the natural P-Pan-CMP intermediate. P-CJ-CMP is a tight-binding inhibitor that prevents further activity of PPCS.^15^ **C)** Bisubstrate mimics of the PPCS reaction intermediate can be formed if pantothenoyl-cytidylate (Pan-CMP, **12**) analogues, in which the reactive acyl phosphate is replaced by a stable linked 1,4-disubstituted triazole, is metabolically activated by PanK to form P-Pan-CMP mimics (**13**). **D)** Alternatively, bisubstrate mimics such as P-CJ-CMP can also be introduced as a masked prodrug (**14**), in which the polar groups are esterified to lipophilic groups that promote cell permeability. After uptake, these are cleaved by intracellular esterases to yield the active inhibitor or its precursor, such as P-CJ (**10**).

While previous studies have identified inhibitors of CoaBC’s PPCS activity through screening efforts,^17,18^ CJ-15,801’s mechanism of action (MoA) offers an alternative approach to PPCS inhibitor discovery.^15^ In the native PPCS reaction, the PanK product 4′-phosphopantothenate (P-Pan, **5**) is activated with CTP to form 4′-phosphopantothenoyl-cytidylate (P-Pan-CMP, **6**), a reactive intermediate that subsequently serves as acyl donor in the reaction with l-cysteine to form 4′-phosphopantothenoylcysteine (P-PanCys, **7**) (Figure 1A). However, PanK phosphorylation of CJ-15,801 serves as metabolic activation of the natural product; the resulting 4′-phospho-CJ-15,801 (P-CJ, **10**) reacts with CTP to form 4′-phospho-CJ-15,801-cytidylate (P-CJ-CMP, **11**), which acts as a tight-binding bisubstrate inhibitor that resists the reaction with l-cysteine, preventing further catalysis (Figure 1B). Importantly, metabolic activation by PanK also serves to introduce selectivity; CJ-15,801 inhibits only *S. aureus* since only its PanK has been found to accept CJ-15,801 as a substrate, allowing for the conversion to **10**. Mechanistically, the Pan analogue hopantenate (HoPan) follows a similar strategy to inhibit the human PPCS, although once phosphorylated it forms an unproductive enzyme–substrate complex.^19^

PPCS’s inhibition by nonreactive bisubstrate reaction intermediate mimics suggests a compelling strategy for the rational design of new PPCS inhibitors, one that has previously been successfully applied to other targets—including targets in *S. aureus* and Mtb—with similar reaction mechanisms.^20^ This would require the preparation of P-Pan-CMP mimics (**13**) through the linking of structural analogues of the P-Pan and CMP moieties with a stable linker. While the *N*-acyl sulfamoyl group has been a popular choice in this regard, in some instances it was found to negatively affect the compounds’ cell permeability and pharmacokinetic properties.^21^ We therefore considered creating bisubstrate mimics by using a 1,2,3-triazole as the linker, prepared by the copper-catalyzed azide–alkyne cycloaddition (CuAAC, aka click reaction) to couple the Pan and CMP moieties (Figure 1C).

Apart from the choice of linker, the 4′-phosphate of the P-Pan-CMP mimics also presents a challenge for the design of cell permeable inhibitors. Here again, CJ-15,801’s MoA offers a possible solution, as preparation of pantothenoyl-cytidylate (Pan-CMP) analogues (**12**)—i.e., without the 4′-phosphate—would be more likely to be cell permeable. If these are accepted as alternate substrates by the pathogen’s PanK, this metabolic activation (and potential point of selectivity control) would lead to the formation of the active inhibitor, i.e., the P-Pan-CMP mimic (Figure 1C). Importantly, the Mtb PanK is a type I PanK (*Mt*PanK_I_) similar to the one present in Escherichia coli, and these—as well as *S. aureus*’s type II PanK (*Sa*PanK_II_)—have been shown to act on a variety of Pan analogues.^22–24^ In fact, it has been reported that the Pan analogue pantothenol inhibits Mtb’s CoaBC (*Mt*CoaBC) following phosphorylation by *Mt*PanK_I_.^25^ We therefore considered it likely that both *Mt*PanK_I_ and *Sa*PanK_II_ would accept the Pan-CMP analogues as substrates, converting them into metabolically active inhibitors.

If PanK failed to perform the necessary metabolic activation step, an alternative strategy for introduction of the phosphorylated inhibitors in a cell permeable form would be as a masked prodrug (**14**) (Figure 1D). Specifically, in these compounds the charged phosphate is masked by derivatization with lipophilic ester groups that are taken up into the cell and then cleaved by endogenous esterases, releasing the active inhibitor.^26^ This approach has been successfully applied to improve the permeability of the highly polar antimicrobial agents fosmidomycin and FR900098,^27^ and could similarly be used to introduce PPCS bisubstrate mimic inhibitors or their precursors, such as P-CJ (**10**).

Building on this knowledge, we set out in this study to explore the potential of bisubstrate mimics of the P-Pan-CMP intermediate as inhibitors of the PPCS activity of both *Mt*CoaBC and *Sa*CoaBC, and to specifically evaluate whether the introduction of the 4′-phosphate by metabolic activation or as a masked prodrug is more likely to yield cell permeable growth inhibitors with potential as new antibacterial agents.

## Results and Discussion

### P-CJ (10) Inhibits *Mt*CoaBC with the Same MoA as for *Sa*CoaBC—Allowing It to Be Used as Common Reference Inhibitor

Our previous report of the inhibition of *Sa*CoaBC’s PPCS activity by CJ-15,801 (**4**) uncovered the requirement for its metabolic activation by *Sa*PanK_II_ to form P-CJ (**10**) (Figure 1B).^15^ However, the same study also reported that while E. coli’s type I PanK (*Ec*PanK_I_) does not phosphorylate CJ-15,801, P-CJ does inhibit the E. coli CoaBC (*Ec*CoaBC) by the same MoA, (i.e., through the *in situ* formation of the bisubstrate inhibitor P-CJ-CMP (**11**)) if P-CJ is supplied directly *in vitro*. Since we found that *Mt*PanK_I_ also does not act on CJ-15,801 (data not shown), we set out to characterize the extent of P-CJ-CMP’s inhibition of *Mt*CoaBC’s PPCS activity if P-CJ is directly introduced to the enzyme in a similar manner.

First, we determined *Mt*CoaBC’s *K*_M_ for P-Pan at saturating CTP concentrations using a coupled assay that links the production of pyrophosphate (the product of the reaction of P-Pan with CTP) with the oxidation of NADH, which can be followed spectrophotometrically at 340 nm.^15^ The resulting activity profile (Figure S1A)—obtained by plotting the initial rates at each substrate concentration—was fit to the Michaelis–Menten equation (eq 1), giving a *K*_M_ value of 33.2 ± 4.2 μM. This value is used in the calculation of the *K*_*i*_, since P-CJ competes with P-Pan for binding.

Next, the PPCS activity of 19.1 nM *Mt*CoaBC was assayed by measuring the formation of pyrophosphate over time at a fixed P-Pan concentration (250 μM) in the presence of increasing concentrations of P-CJ (**10**). The resulting set of progress curves (Figure 2A) was used to calculate and plot the fractional activity (*v*_i_/*v*_0_) at each concentration of P-CJ. P-CJ-CMP was previously shown to act as a tight-binding inhibitor of *Sa*CoaBC; such inhibitors exhibit increasing IC_50_ values as the total enzyme concentration is increased (at a fixed substrate concentration) as they generally interact with their enzymes in nearly stoichiometric fashion.^28^ Performing the assay at an additional *Mt*CoaBC concentration (9.6 nM) (Figure S1B) gave two fractional activity curves that clearly have different IC_50_ values (Figure 2B), indicating that once formed, P-CJ-CMP also acts as a tight-binding inhibitor of *Mt*CoaBC. By fitting the Morrison equation for tight-binding inhibitors (eq 2) to the data for each curve separately, the respective *K*_*i*_^app^ values were obtained; these were converted to *K*_*i*_ values using the Cheng-Prusoff equation appropriate for competitive inhibitors (eq 3) and the determined *K*_M_ value. The average *K*_*i*_ value calculated in this manner from the two plots was 58.7 nM. Using the same methodology to characterize P-CJ-CMP’s inhibition of *Sa*CoaBC gave an average *K*_*i*_ value of 32.3 nM (Figure S1C–E). Although this is higher than the previously reported value of 13 nM,^15^ the difference can be reasonably attributed to the difficulty in accurately determining the concentration of active protein used in the analyses.^28^

**Figure 2:**
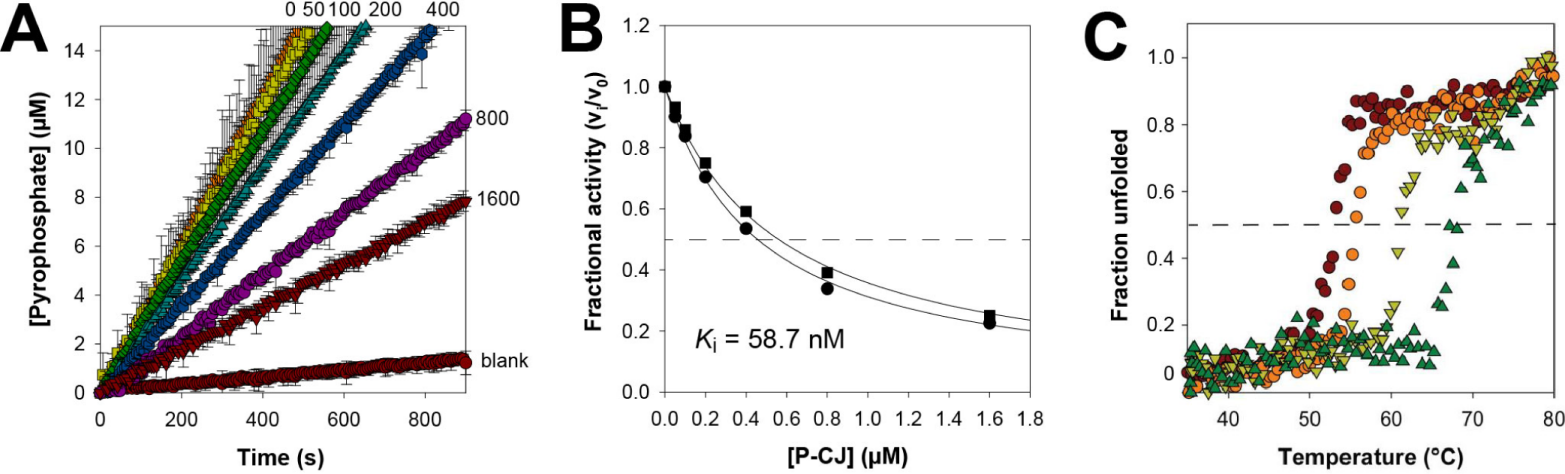
Characterizing the inhibition of*Mt*CoaBC by P-CJ-CMP (11). **A)** Progress curve analysis for the inhibition of 19.1 nM *Mt*CoaBC in the presence of increasing concentrations of P-CJ (**10**), indicated in nM next to each curve. Reaction rates were determined in triplicate at each [P-CJ]; the symbols show the average rate and the error bars indicate the standard deviation of the triplicate values. **B)** The fractional activity of *Mt*CoaBC as tested at 19.1 nM (■) and 9.6 nM (●) as a function of [P-CJ]. The solid line represents the data fit to the Morrison equation (eq 2). **C)** Normalized heat-induced protein melting curves for *Mt*CoaBC determined by following the changes in the protein’s secondary structure by circular dichroism (CD) spectroscopy. The four curves represent from left to right: the apo protein (10 μM); 10 μM protein with 150 μM MgCTP; 10 μM protein with 150 μM MgCTP and 150 μM P-Pan (**5**); and 10 μM protein with 150 μM MgCTP and 150 μM P-CJ (**10**).

Finally, the change in *Mt*CoaBC’s melting temperature (*T*_m_)—measured by following the heat-induced change in its secondary structure using circular dichroism (CD) spectroscopy—was used to quantify the relative impact that the binding of a natural ligand or inhibitor has on the protein’s stability. The same general trend was observed as seen in our previous studies of *Ec*CoaBC,^15^ i.e., the addition of the natural substrate CTP resulted in a small stabilization, the formation of the natural reaction intermediate P-Pan-CMP (**6**) a larger shift (Δ*T*_m_ = 8.0 °C), and the formation of the tight-binding bisubstrate mimic P-CJ-CMP (**11**) an even larger shift (Δ*T*_m_ = 15.3 °C). Comparatively, the shift in the presence of the inhibitor is bigger in the case of *Sa*CoaBC (Δ*T*_m_ ∼ 20 °C) (Figure S1F); this corresponds with the lower *K*_*i*_ value determined in its case. Taken together, these results confirm that like *Sa*CoaBC, *Mt*CoaBC is also susceptible to inhibition by P-CJ-CMP (**11**) formed *in situ* by reaction of P-CJ (**10**) with CTP, and that this can be used as reference for the development of bisubstrate reaction intermediate mimics as new inhibitors of the PPCS activity of these CoaBC enzymes.

### Design and Synthesis of Triazole-Linked Bisubstrate PPCS Inhibitors

Our choice of the 1,2,3-triazole moiety as a nonreactive bioisostere to replace the reactive acyl phosphate of **6** was based on its previous successful use in the preparation of *S. aureus* biotin ligase inhibitors.^29^ Not only does the triazole contribute the necessary hydrogen bond acceptors, it also provides a convenient route by which to couple the nucleotide and pantoyl/pantothenoyl moieties, i.e., by means of click chemistry. We proposed to achieve this by using known 5′-azido-5′-deoxycytidine (**19**)^30^ and by simply incorporating the alkyne into the pantothenoyl moiety by generating its propargyl ester (**17a**) and amide (**17b**) analogues (Scheme 1). However, we considered that the placement of the triazole in the resulting linked Pan-CMP mimics **12a** and **12b** may push the pantothenoyl moiety too far from the nucleotide, and lead to suboptimal binding interactions in the active site. Consequently, we decided to broaden the scope of inhibitor candidates by replacing the pantothenoyl group’s β-alanine (β-Ala) moiety with terminal alkyne amines of varying lengths (**16**) to prepare pantoylamides **21** from pantolactone **20**. Click cycloaddition of these amides with **19** then gave the Pan-CMP mimics **12c–e**.

**Scheme 1:**
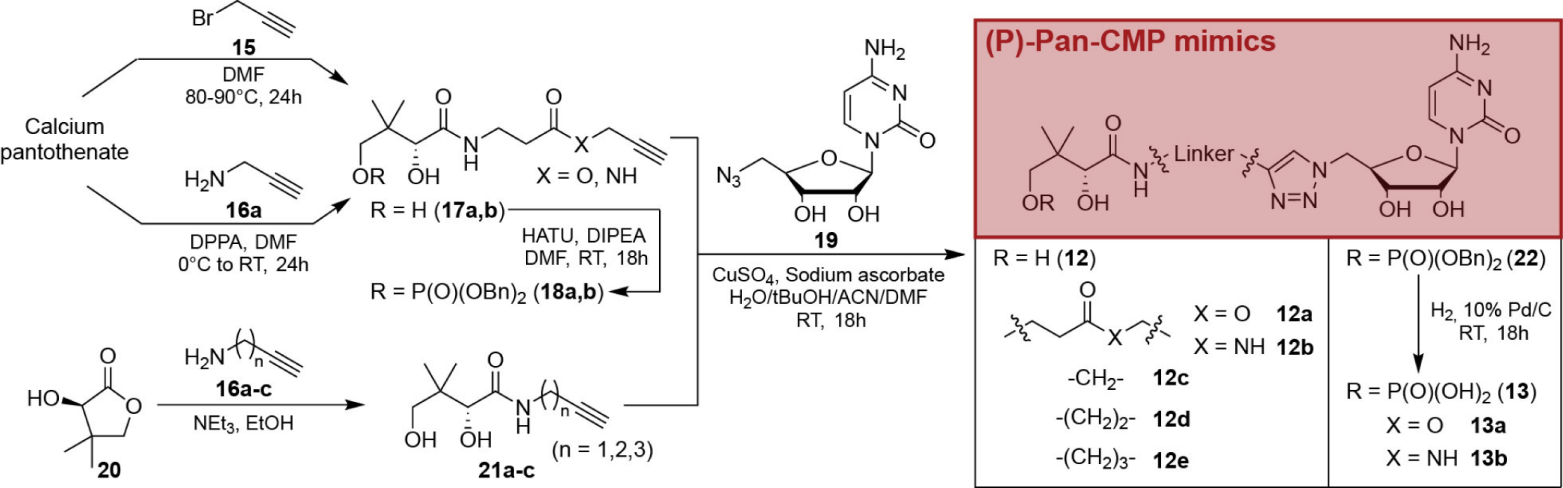
Synthesis of Pan-CMP Bisubstrate Mimics Designed for Metabolic Activation^a^

a Five Pan-CMP mimics (**12**) were prepared by coupling the pantothenate propargyl ester **17a**, pantothenate propargyl amide **17b**, or the terminal alkyne substituted pantoamides **21a–c** to 5′-azido-5′-deoxycytidine **19** by click cycloaddition, yielding analogues bearing a 1,2,3-triazole linker. The phosphorylated versions (P-Pan-CMP mimics **13**) of the ester and amide were similarly prepared by first introducing the dibenzyl phosphates from **18a** and **18b**, and deprotecting the phosphate by catalytic hydrogenation after the click coupling to give **13a** and **13b**.

### Kinetic Evaluation of the Pan-CMP Bisubstrate Mimics for Metabolic Activation and PPCS Inhibition

With the Pan-CMP mimics **12** in hand, we first set out to test whether metabolic activation by the PanKs of the target organisms would take place. For these assays, compounds were offered as substrates to either *Sa*PanK_II_ or *Mt*PanK_I_ at a concentration that would be saturating for Pan (**2**), i.e., 400 μM, or ∼ 20-fold the *K*_M_ for Pan. Activity was monitored continuously using the standard pyruvate kinase (PK)/lactate hydrogenase (LDH) kinase assay that couples the production of ADP during phosphorylation to the oxidation of NADH, which is tracked spectrophotometrically.^31^ The resulting initial rates, reported as a percentage of the rate measured for Pan, are shown in Figure 3A. *Sa*PanK_II_ showed the best activity toward pantothenoyl ester **12a** and extended pantoamide **12e**, with 58.3 ± 2.4% and 60.4 ± 0.5% of the activity of Pan, respectively. It was nearly inactive toward pantoamide **12c** with the methylene linker, had very little activity toward pantothenamide **12b** and had intermediate activity with pantoamide **12d** with the ethylene linker. *Mt*PanK_I_, in contrast, showed very little activity toward any of the pantoamides, had some (∼15%) activity toward pantothenoyl ester **12a** and showed the best activity toward pantothenamide **12b** (44.6 ± 0.8%). This suggests that *Mt*PanK_I_’s substrate specificity is more stringent, considering that it only recognizes mimics that retain the full pantothenoyl moiety. The distinct results obtained for the two PanKs clearly demonstrate that any structure–activity relationship (SAR) analysis of the metabolic activation of Pan-CMP mimics would have to be done for each PanK individually.

**Figure 3:**
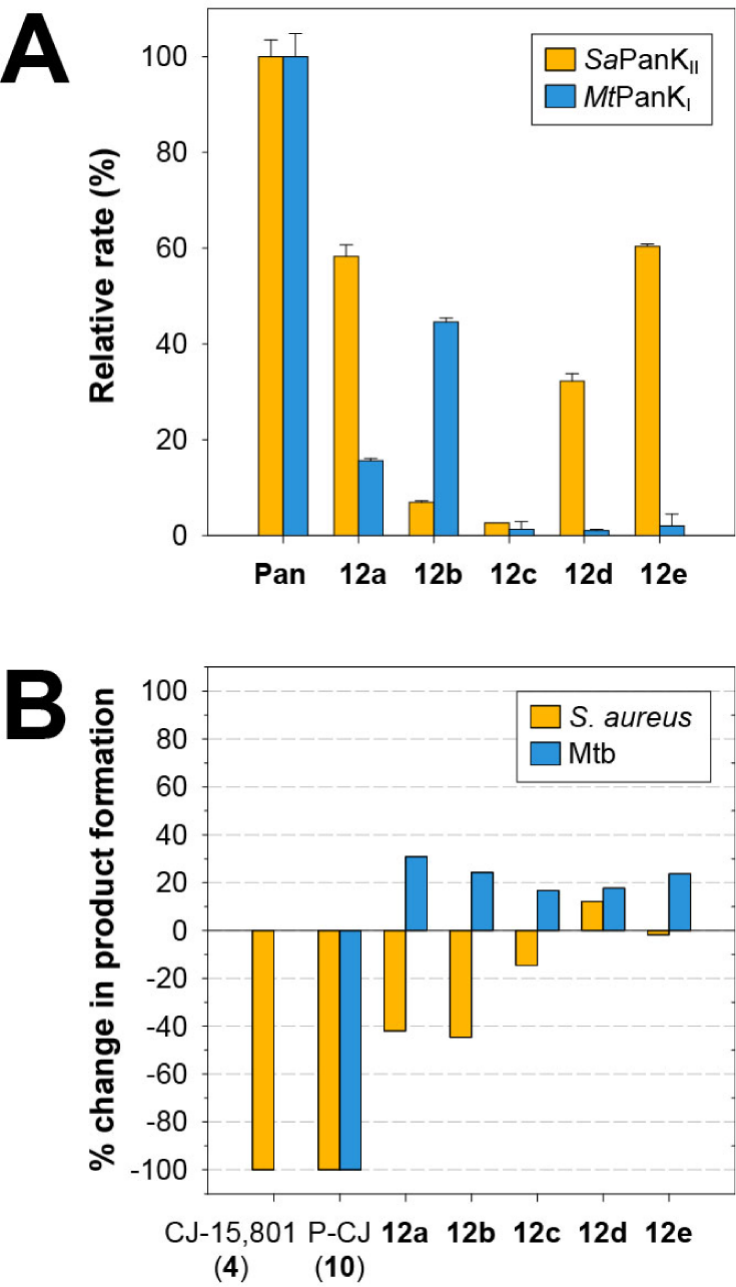
Metabolic activation of the Pan-CMP mimics and their subsequent inhibition of PPCS activity. **A)** *In vitro* activity of *Sa*PanK_II_ and *Mt*PanK_I_ toward the indicated Pan-CMP mimics (**12**), reported as a percentage of the activity measured with Pan (**2**) in a bar graph according to the shown legend. All activities were determined by measuring the initial rate of 0.01 mg/mL enzyme toward 400 μM of substrate using the pyruvate kinase/lactate dehydrogenase kinase coupled kinase assay. The ATP concentration was 1.5 mM and 3.0 mM for *Sa*PanK_II_ and *Mt*PanK_I_ respectively. The data represent the average of technical triplicates, with the errors denoting the standard deviation. **B)** Inhibition of the PPCS activity of CoaBC, determined in an *in vitro* systems-based assay by measuring the change in amount of P-PantSH (**8**) formed from the PPCS substrates in the presence of 400 μM of the indicated compounds. The result is shown as the percentage change in the amount of P-PantSH (**8**) formed relative to a reference reaction containing 1 mM Cys, 1 mM CTP and either 0.01 mg/mL *Sa*PanK_II_, 0.09 mg/mL *Sa*CoaBC and 1.5 mM ATP (*S. aureus* system) or 0.01 mg/mL *Mt*PanK_I_, 0.022 mg/mL *Mt*CoaBC and 3.0 mM ATP (Mtb system). Reaction mixtures were first incubated with the indicated compound for 15 min before initiating the reaction by addition of 500 μM Pan (**2**). The amount of P-PantSH (**8**) formed was measured after 10 min in the *S. aureus* system; for the Mtb system the combined amount of P-PanCys (**7**) and P-PantSH (**8**) formed was measured after 20 min.

Next, we wanted to determine whether the Pan-CMP mimics, once metabolically activated (i.e., phosphorylated to form the P-Pan-CMP mimics **13**), inhibit the PPCS reaction of the *Sa*CoaBC and *Mt*CoaBC proteins. For this, a systems-based assay was used in which the PanK and CoaBC proteins are combined and supplied with all the required substrates and cofactors, except Pan (**2**). Next, each of the Pan-CMP mimics **12** was added to allow for their metabolic activation by the relevant PanK without any interference by its native substrate. Finally, after 15 min of preincubation, Pan was added and the PanK/CoaBC enzyme mixture was incubated for 10 min (for *Sa*PanK_II_ and *Sa*CoaBC) or 20 min (for *Mt*PanK_I_ and *Mt*CoaBC) before quantifying the amount of P-PantSH (**8**) (the product of the PPCDC activity of CoaBC, see Figure 1A) formed. This was done by using a published HPLC method that relies on the precolumn derivatization of all thiolated compounds to yield fluorescent adducts.^32^ For these tests, CJ-15,801 (**4**) and P-CJ (**10**) were used as controls for inhibition: the former is expected to be phosphorylated by *Sa*PanK_II_ and once metabolically activated to inhibit the PPCS reaction of *Sa*CoBC completely, while no inhibition is expected in the Mtb system as *Mt*PanK_I_ does not act on CJ-15,801. However, full inhibition should occur with the preactivated P-CJ (**10**) in both systems.

The results, reported as the percentage change in the amount of product formed in a reference condition with only Pan and no inhibitor, show that the inhibition controls perform as expected, with full inhibition observed for both CJ-15,801 (**4**) and P-CJ (**10**) in the *S. aureus* system, and only for P-CJ (**10**) in the Mtb one (Figure 3B). However, none of the bisubstrate mimics showed similar potency, with only the two inhibitors containing the full pantothenoyl moiety (**12a** and **12b**) reducing the amount of product formed by the *S. aureus* system more than 40%. This result is despite the good metabolic activation that was seen for some of the mimics—such as for **12e** with *Sa*PanK_II_—again highlighting the importance of considering both the efficiency of metabolic activation and the potency of inhibition when developing inhibitors with such a MoA.

Interestingly, all five bisubstrate mimics had an apparent stimulating effect on the Mtb system, as the amount of product formed was ∼ 15–30% more than in the reference condition with Pan (**2**) alone. This can be explained in light of the structure of the homologous *Mycobacterium smegmatis* CoaBC, which exists as a dodecameric protein with putative allosteric binding sites.^17^ While it was proposed that binding to these sites causes inhibition of its PPCS activity, it is possible that the bisubstrate mimics have an alternative effect—such as the promotion of cooperative binding—that results in the observed stimulation of activity.

### Probing P-Pan-CMP Bisubstrate Mimic Binding to CoaBC Using Differential Scanning Fluorimetry (DSF)

While the kinetic analyses provide direct insight into the inhibition of PPCS activity by the bisubstrate mimics, such analyses are complex and time-consuming to execute. It would be ideal if a simpler, direct binding assay could be used as a primary screen of potential PPCS inhibitors. We therefore set out to use differential scanning fluorimetry (DSF) to determine if the results of the kinetic analyses could be recapitulated in a simpler direct binding assay. We chose to use DSF for this analysis instead of CD spectroscopy as was done initially (Figure 2C) because the smaller assay format requires less protein and is amenable to higher sample throughput; it has also been used successfully for inhibitor characterization studies of the PPCS activity of *Mt*CoaBC.^18^ However, to establish if DSF gives results that are comparable to the CD-based analysis, the thermal melt curves of *Sa*CoaBC and *Mt*CoaBC were determined with DSF using conditions similar to those used previously, i.e., protein in the presence of a 10-fold excess of CTP (*Apo*; the reference state), or protein with a 10-fold excess of mixture of P-CJ (**10**) and CTP—allowing for the formation of the active inhibitor, P-CJ-CMP (**11**). In addition, we also synthesized the phosphorylated versions of the two Pan-CMP mimics (**12a** and **12b**) that gave the best results in the kinetic analysis for their inclusion in the binding evaluation. P-Pan-CMP mimics **13a** and **13b** were prepared from **17a** and **17b** by coupling with dibenzyl phosphate to give **18a** and **18b.** Click cycloaddition yielded **22a** and **22b**, after which the benzyl protecting groups were removed to give the desired compounds (Scheme 1).

The results show that the DSF-derived thermal melt curves give the same relative result as those obtained using CD spectroscopy, i.e., the P-CJ-CMP inhibitor formed *in situ* resulted in significant stabilization of both *Sa*CoaBC (Figure 4A) and *Mt*CoaBC (Figure 4B). Moreover, in the case of *Mt*CoaBC, the absolute *T*_m_ values also closely correspond to those obtained using CD spectroscopy (compare Figure 2C). However, in the case of *Sa*CoaBC, the determined *T*_m_ values were much lower, being reduced by 20 °C or more (compare Figure S1F). This suggests that DSF, and specifically the binding of the SYPRO Orange dye used in this analysis, may have a destabilizing effect on the structure of the *Sa*CoaBC protein, and that results obtained using this method should be interpreted with care. However, we were pleased to observe that both proteins were stabilized by the addition of the P-Pan-CMP mimic **13a**, with *T*_m_ values intermediate between that obtained with CTP alone and with the P-CJ-CMP inhibitor formed *in situ*. For *Sa*CoaBC, this corresponds to the result obtained in the kinetic analysis and indicates that in its case a simple binding analysis could be used as a primary evaluation of the potency of bisubstrate mimic inhibitors. In contrast, the observation of **13a**’s binding to *Mt*CoaBC only serves to confirm that the stimulation of activity observed for **12a**, which is presumably phosphorylated *in situ* to **13b**, is not an artifact but follows from the interaction of the bisubstrate mimic with the protein.

**Figure 4:**
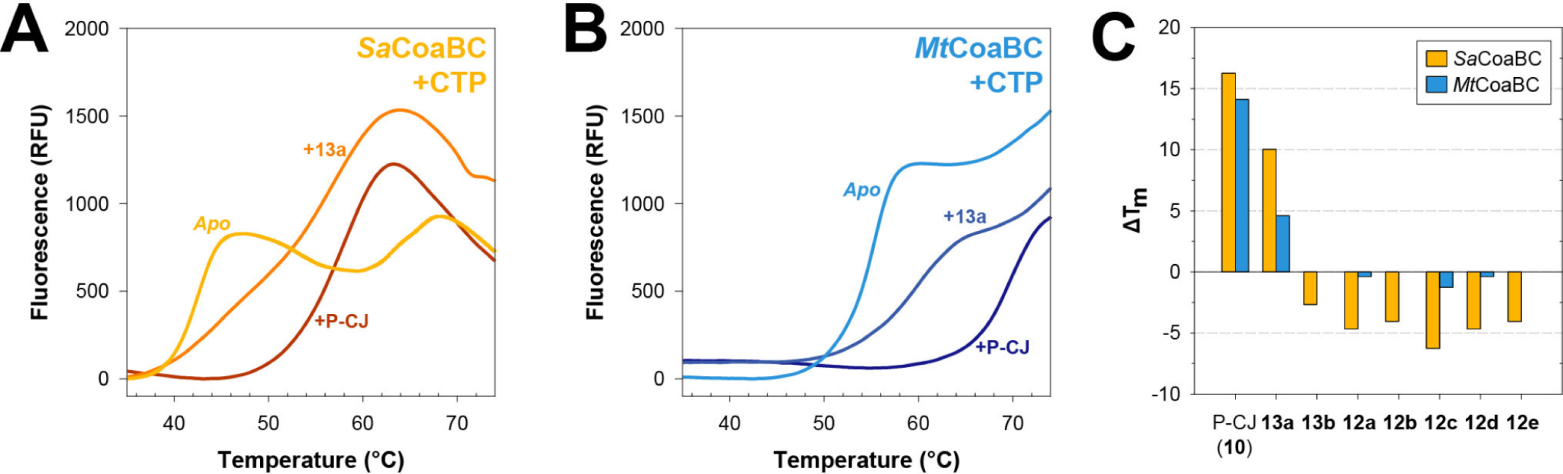
Binding analysis of P-Pan-CMP bisubstrate mimics to CoaBC using differential scanning fluorimetry (DSF). **A)** Thermal melt curve of 10 μM *Sa*CoaBC and 100 μM CTP alone (*Apo*), 100 μM CTP incubated with 100 μM P-CJ (**10**) (allowing for formation of P-CJ-CMP (**11**) *in situ*) or 100 μM CTP and 100 μM of the P-Pan-CMP mimic **13a**. **B**) As for Panel A, but for *Mt*CoaBC. **C**) Change in melting temperature (Δ*T*_m_) of a mixture of each of the CoaBC proteins and 100 μM CTP (*Apo*; reference state) compared to when the same mixture is incubated with 100 μM of the indicated compound.

Finally, we used DSF to determine the change in the *T*_m_ values of both proteins in the presence of 10-fold excess CTP upon addition of P-CJ (**10**) as reference, or either of the two synthesized P-Pan-CMP mimics (**13a** and **13b**) and each of the Pan-CMP mimics (**12a–e**). This was done to confirm the requirement of the 4′-phosphate for binding, and by implication the need for its installation by metabolic activation or some other mechanism. The results show that, as expected, none of the unphosphorylated Pan-CMP mimics have stabilizing binding interactions with either of the CoaBC proteins when present at 100 μM (i.e., in 10-fold excess) (Figure 4C). In the case of *Sa*CoaBC, the interactions are in fact destabilizing, i.e., the Pan-CMP mimics preferentially bind to a non-native conformational state of the protein, shifting the equilibrium between the folded and unfolded protein states toward the latter.^33,34^ It is possible that this outcome is also an effect related to the impact of SYPRO Orange binding to the protein, although similar destabilizing effects (albeit smaller in magnitude) are seen with *Mt*CoaBC. Unexpectedly, the same effect is also seen with the P-Pan-CMP mimic **13b**. To determine whether this is possibly due to degradation of the compound, we repeated the binding analysis at a higher concentration (1.0 mM). At this concentration, both **13a** and **13b** showed stabilizing effects, increasing the *T*_m_ of the *Mt*CoaBC/CTP mixture by 10.9 °C and 6.3 °C, respectively (data not shown). This confirms that the low signal observed for **13b** tested at 100 μM is not due to loss of its phosphate, but to the rank order of binding affinity for the two phosphorylated mimics (**13a** > **13b**). It also highlights that the dynamic range of the binding analysis may be increased by using higher ligand concentrations in the DSF experiment.

### Design and Synthesis of PPCS Bisubstrate Mimic Prodrugs Based on P-CJ

The results of the kinetic and binding analyses of the Pan-CMP analogues indicated that while the design of such bisubstrate mimics as inhibitors of the PPCS activity of CoaBC proteins is possible, their development will require a multifaceted approach that optimizes both their metabolic activation by PanK and their inhibitory binding interactions with CoaBC. Consequently, while the metabolic activation by PanK was initially considered to be potentially useful in increasing selectivity, retaining it would involve significant investment in the development process of such inhibitors. We therefore decided to determine if it would be possible to also prepare PPCS bisubstrate mimic inhibitors by bypassing the need for metabolic activation. To achieve this, we synthesized membrane-permeable prodrugs of P-CJ (**10**) as precursors of the bisubstrate mimic P-CJ-CMP (**11**), which clearly shows exceptional inhibition of both CoaBC proteins (Figure 3B). Specifically, P-CJ’s phosphate group was masked as pivaloyloxymethyl (POM) esters, while its carboxylate was masked using one of three esters: POM (**14a**), (5-methyl-2-oxo-1,3-dioxol-4-yl)­methyl (**14b**), *m*-trifluoromethylbenzyl (**14c**) or left unprotected (**14e**, prepared by deprotection of allyl ester **14d**) (Scheme 2). The protected precursors to these prodrugs (**25a–d**) were prepared using a Buchwald-Hartwig type amination of acetonide-protected d-pantoamide (**24**) with appropriate bromoacrylic ester precursors (**23a–d**), catalyzed by Pd­(OAc)_2_/Xantphos and cetyltrimethylammonium bromide (CTAB) in the presence of potassium carbonate.^35^ Following deprotection of the acetonide, phosphorylation of **26a–d** with bis­(POM)-phosphoryl chloride (**27**)^36^ gave the desired products **14a–c**. Furthermore, allyl ester **14d** was phosphorylated using bis­(POM)-phosphoric acid (**28**)^36^ and HATU as coupling agent.

**Scheme 2:**
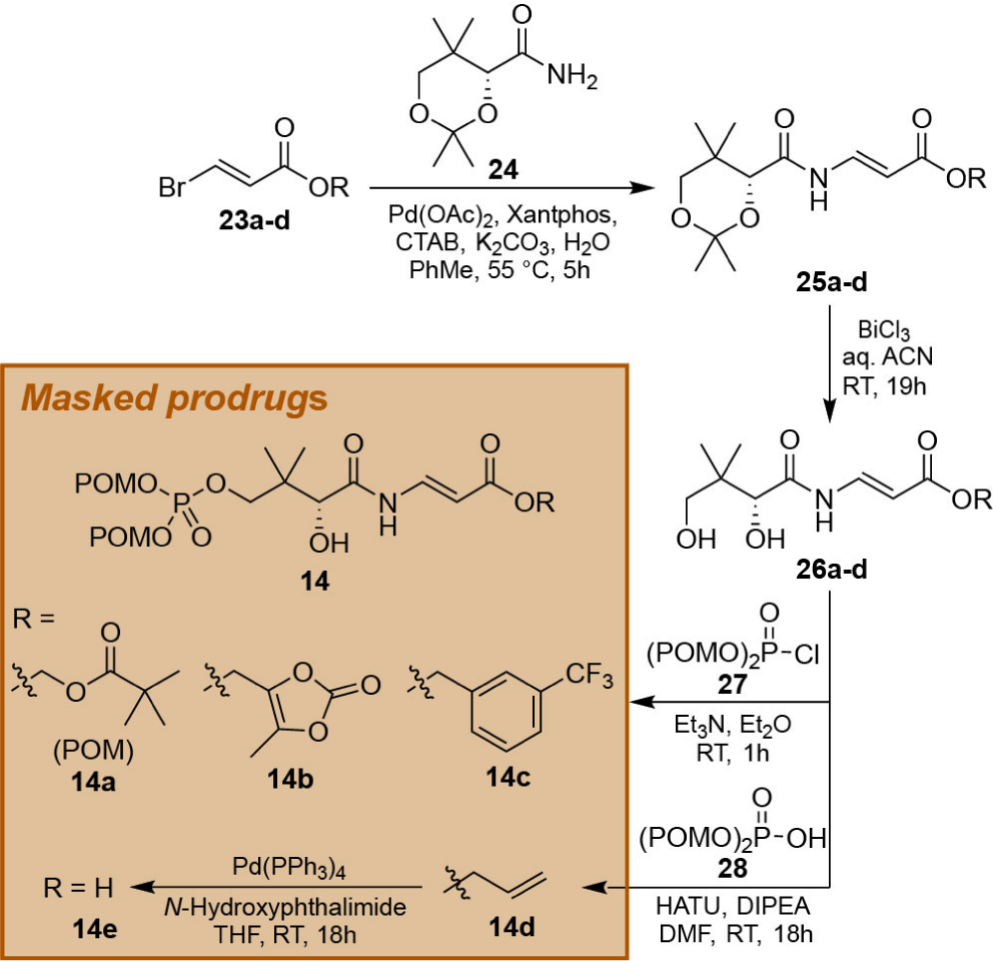
Synthesis of Masked P-CJ Prodrugs as Precursors of the P-CJ-CMP Bisubstrate Mimic^a^

a Four masked prodrugs of P-CJ (**10**) were prepared by Pd-catalyzed amination of pantoamide **24** with bromoacrylic esters **23a–d** to give the enamides **25a–d**. Acetonide deprotection and phosphorylation with bis­(POM)-phosphoryl chloride (**27**) gave the desired products **14a–c**. Allyl ester **14d** was phosphorylated using bis­(POM)-phosphoric acid (**28**) and HATU, and was subsequently deprotected to give the free acid **14e**.

### Evaluation of Pan-CMP Bisubstrate Mimics and Masked P-CJ Prodrugs as Whole Cell Growth Inhibitors of *S. aureus* and Mtb

Regardless of the *in vitro* results, the crucial test of both the Pan-CMP bisubstrate mimics **12** and masked P-CJ prodrugs **14** is their ability to inhibit the growth of *S. aureus* and Mtb. Unfortunately, none of the Pan-CMP bisubstrate mimics **12** showed inhibition of *S. aureus* at concentrations up to 100 μM, even when grown in minimal medium, i.e., when no exogenous Pan (**2**) is available to antagonize their inhibitory potency (Table 1). This suggests that, assuming the compounds are taken up, their metabolic activation does not proceed sufficiently fast to allow for the formation of effective inhibitors inside the cell. In contrast, the masked P-CJ prodrugs **14a** and **14b** did show inhibition of *S. aureus* when grown in rich medium (tryptic soy broth), with MIC values of ∼ 50 μM and ∼ 100 μM, respectively. This is much higher than the MIC value of ∼ 5 μM determined for CJ-15,801 (**4**) under the same conditions, suggesting that its metabolic activation by *Sa*PanK_II_ proceeds faster than the enzymatic unmasking of the protecting groups of **14**, or that the uptake of the latter compound is slower than that of free CJ-15,801 (**4**). The prodrugs **14c** and **14e** were inactive at the highest concentration tested. Considering this, and the fact that the dose–response curves of the inhibition shown by **14a** and **14b** have different slopes (Figure S2), it is likely that the rate of unmasking of the protecting groups is a crucial determining factor in the observed potency.

**Table 1:** Minimum Inhibitory Concentrations (MICs) of Whole Cell Growth Inhibition of *S. aureus* and Mtb by Pan-CMP Bisubstrate Mimics **12** and Masked P-CJ Prodrugs **14**

		**MIC (μM)** ^a^				
	*S. aureus* **RN4220**	*S. aureus* **Xen29** ^b^	**Mtb H37RvMA** ^37^			**Mtb H37RvMA** *coaBC* **cKD**
**Compound/** *Medium* ^c^	*Minimal medium*	*TSB*	*7H9*	*GAST/Fe*	*Acetate*	*Acetate*
**12a–e**	>100	ND^d^	>1000	∼4000	∼4000	∼4000
**14a**	ND	∼50	∼500	31.25	62.5	15.65
**14b**	ND	∼100	∼500	62.5	250	125
**14c**	ND	>200	>500	125	62.5	62.5
**14e**	ND	>400	>250	ND	ND	ND
CJ-15,801 **(4)**	ND	∼5	ND			

a MIC values in μM determined for the indicated strain of *S. aureus* or Mtb when grown in the indicated growth medium.

b Masked P-CJ prodrugs **14** were evaluated against *S. aureus* Xen29 (a bioluminescent derivative of methicillin-susceptible *S. aureus* strain ATCC 12600).

c Growth media are TSB, tryptic soy broth; 7H9, Middlebrook 7H9 broth supplemented with Middlebrook albumin-dextrose-catalase (ADC) enrichment, 0.2% glycerol, and 0.05% Tween-80; GAST/Fe, glycerol-alanine salts with iron minimal medium; Acetate, Middlebrook 7H9 broth supplemented with 10 mM acetate as the sole carbon source.

d ND, not determined.

Next, we considered the whole cell inhibition of Mtb grown in various media. These included Middlebrook 7H9 supplemented with Middlebrook Albumin Dextrose Catalase (ADC) and glycerol as carbon source, GAST/Fe (glycerol-alanine salts with iron) minimal media and growth medium with acetate as the main carbon source. The difference in carbon sources in these media was considered because of the importance of CoA in energy metabolism; for example, under low energy conditions CoA pools are affected and dependence on growth factors such as CoA may be increased.^38^ We therefore wanted to compare the impact of our compounds on Mtb grown on a high energy carbon source (glycerol) to a low energy carbon source (acetate), as well as when a minimal, albumin-free medium is used.

As in the case of *S. aureus,* none of the Pan-CMP bisubstrate mimics **12** showed any activity (Table 1). However, this was expected because of the poor metabolic activation of these compounds by *Mt*PanK_I_ (Figure 3A). In contrast, the masked P-CJ prodrugs **14** all showed some activity in either GAST/Fe minimal medium or when grown in the presence of acetate as carbon source, with the tris­(POM)-protected **14a** showing the best potency in both media, with MIC values of 30 μM and 62.5 μM, respectively. More importantly, when evaluated against a *coaBC* conditional knockdown mutant (hypomorph) grown in acetate medium, the potency of both **14a** and **14b**, but not **14c**, increased (Table 1). In the hypomorph *coaBC* expression is under control of a tetracycline-regulated promoter; addition of increasing concentrations of the inducer, anhydrotetracycline (ATc), therefore causes progressive transcriptional silencing of this gene, leading to lower levels of *Mt*CoaBC. The hypersensitivity observed toward **14a** and **14b**, therefore, confirms that these compounds act on target, inhibiting *Mt*CoaBC and the CoA pathway.

## Conclusion

The validation of CoaBC as a bactericidal target in Mtb raised the profile of CoA biosynthesis and the potential of this particular protein for antibacterial drug development. This has led to a high-throughput screen (HTS) being conducted to identify inhibitors of its PPCS activity, which yielded a hit derived from a scaffold built on the structure of edaravone, an amyotrophic lateral sclerosis (ALS) treatment.^18^ This compound inhibits the enzyme with an IC_50_ of ∼ 16 μM and has an MIC of ∼ 26 μM against Mtb grown in 7H9 medium. We considered identifying PPCS inhibitors with at least similar potency using a rational approach by preparing bisubstrate mimics of its reaction intermediate, P-Pan-CMP (**6**), based on this strategy successfully being used on other targets with related MoAs. We chose to design our inhibitors using a 1,2,3-triazole linker, and to promote the introduction of the polar 4′-phosphate through metabolic activation by action of PanK, the first enzyme in the CoA pathway, as this strategy is also used by other substrate-mimicking CoA pathway inhibitors.^3^ Unfortunately, this proved to be a significant challenge as the *Mt*PanK_I_ enzyme was found to be less promiscuous than other bacterial type I PanKs, which resulted in very low levels of activation. In addition, even when phosphorylated and able to bind to their target, the bisubstrate mimics failed to show inhibition of the PPCS activity that rivaled that of the natural product CJ-15,801 (**4**), which forms a bisubstrate mimic inhibitor *in situ*. In fact, for *Mt*CoaBC binding of the bisubstrate mimics unexpectedly seemed to stimulate activity instead. In the end, none of the Pan-CMP bisubstrate mimics showed whole cell inhibition of either *S. aureus* or Mtb. These factors highlight the complexity of developing CoaBC inhibitors.

Consequently, we decided to use the scaffold of the known PPCS inhibitor CJ-15,801, which also requires metabolic activation through phosphorylation, to determine if the essential 4′-phosphate can be preinstalled and introduced as a masked prodrug, as was done for fosmidomycin.^27^ Using this approach we succeeded in designing CoaBC inhibitors that showed modest whole cell activity against both *S. aureus* and Mtb, with the most potent Mtb inhibitor having an MIC very similar to that of the best inhibitor (based on the edaravone scaffold) identified through HTS. Importantly, in the case of Mtb, this activity was confirmed to be on-target, and the potency was found to be the highest when growth occurred when energy sources were limited. This suggests that CoA pathway inhibitors should be evaluated particularly under conditions leading to the metabolic rewiring that places a high demand on CoA utilization.

Taken together, this study shows that while pursuing bisubstrate mimics as inhibitors of CoaBC’s PPCS activity holds promise, a more extensive SAR analysis would be required to determine which structural components contribute most to inhibitory binding interactions. In this context it would likely be fruitful to explore modification or replacement of the cytidine moiety so as to make an SAR analysis more amenable. In addition, other linker options could also be explored. However, the pantothenoyl moiety will have to be retained, as our results also show that its modification is detrimental for binding to CoaBC. Such changes may also promote the ability of the PanKs to perform metabolic activation. Failing this, the use of prodrugs to mask the required 4′-phosphate is clearly also a viable option for the preparation of cell-permeable CoaBC inhibitors as new potential antimicrobial agents.

## Supplementary Material



## Notes

Figures S1 and S2. All materials and methods, including synthetic methods for all synthesized compounds (PDF)
