# Supplementary material for: Metabolic Activation versus Masked Prodrugs: Bisubstrate Mimic Inhibitors of CoaBC’s PPCS Activity in Mycobacterium tuberculosis and Staphylococcus aureus
Source: ACS Infect Dis. 2025 Jun 3;11(6):1508–17. doi: 10.1021/acsinfecdis.5c00047 (PMC12172042; doi:10.1021/acsinfecdis.5c00047)
Supplement: Supplementary file 1 [file id5c00047_si_001.pdf]

Supporting Information

Metabolic Activation vs Masked Prodrugs: Bisubstrate Mimic Inhibitors of CoaBC's PPCS Activity in *Mycobacterium tuberculosis* and *Staphylococcus aureus*

Timothy J. Kotzé[1†], Konrad J. Mostert[1†], Riyad Domingo[1], Xu Wang[2], Wessel J.A. Moolman[1], Hailey S. Butman[2], Abigail Pepin[2], Kyle T. McKay[2], Deon P. Neveling[1], Joanna C. Evans[3], Valerie Mizrahi[3], Willem A.L. van Otterlo[4], Cynthia S. Dowd[2]* & Erick Strauss[1]*

[1]Department of Biochemistry, Stellenbosch University, Stellenbosch, 7600, South Africa.
[2]Department of Chemistry, The George Washington University, Washington DC 20052, USA.
[3]Molecular Mycobacteriology Research Unit, Institute of Infectious Disease and Molecular Medicine and Department of Pathology, Faculty of Health Sciences, University of Cape Town, Observatory, 7925, South Africa.
[4]Department of Chemistry & Polymer Science, Stellenbosch University, Stellenbosch, 7600, South Africa.

[†]These authors contributed equally.

***Contact information**

Erick Strauss (contact corresponding author):
e-mail: estrauss@sun.ac.za

Cynthia S. Dowd (co-corresponding author):
e-mail: cdowd@gwu.edu

SUPPLEMENTARY FIGURES

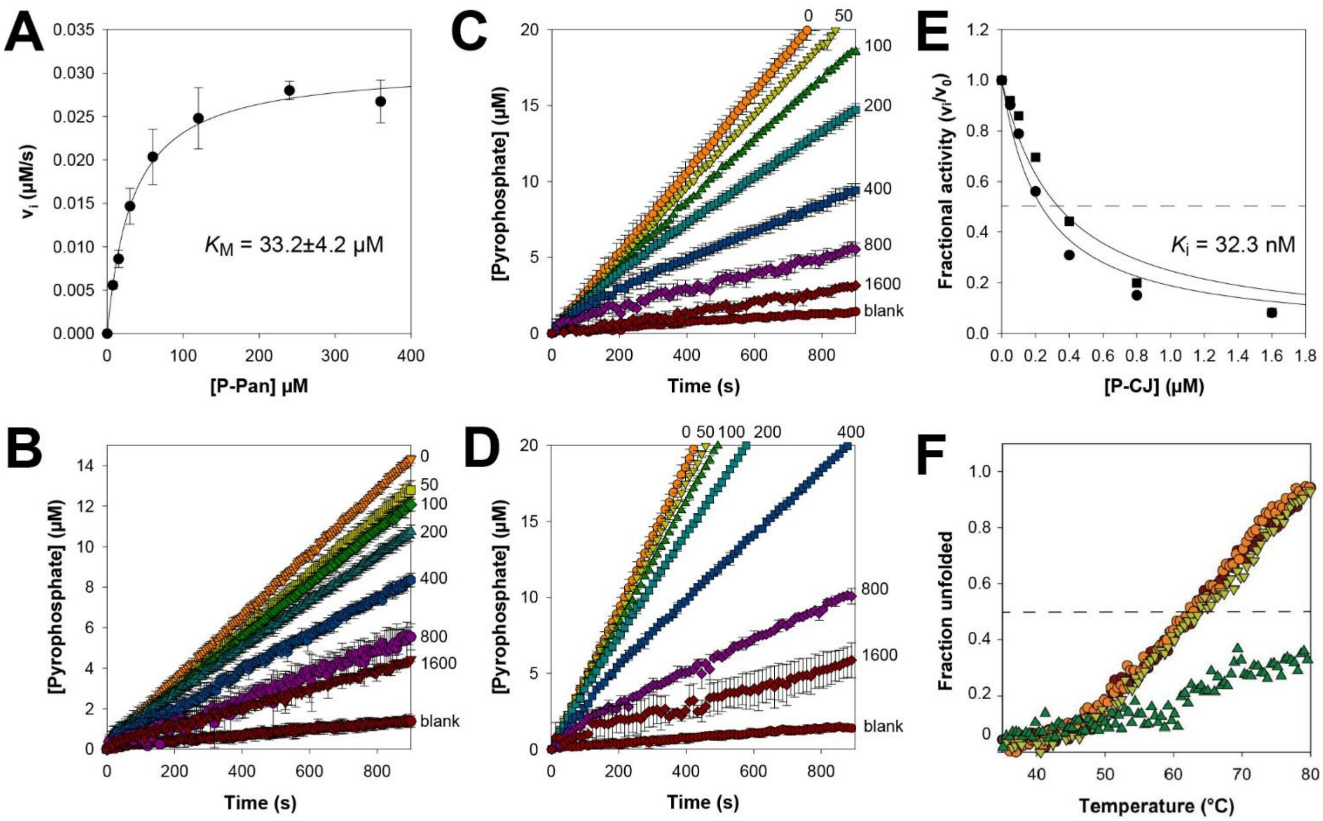

Supplementary Figure S1. Characterizing the inhibition of *Mt*CoaBC and *Sa*CoaBC by P-CJ-CMP (11). A) Activity profile of *Mt*CoaBC in the presence of increasing concentrations of P-Pan (**5**) while maintaining a constant concentration of CTP (1.0 mM) and L-Cys (1.0 mM). Symbols represent the average of initial rates determined in triplicate at each P-Pan concentration; the error bars indicate the standard deviation. The solid line represents the data fit to the Michaelis-Menten equation (Eqn. 1). **B)** Progress curve analysis for the inhibition of 9.6 nM *Mt*CoaBC in the presence of increasing concentrations of P-CJ (**10**), indicated in nM next to each curve. Reaction rates were determined in triplicate at each [P-CJ]; the symbols show the average rate and the error bars indicate the standard deviation of the triplicate values. **C**) As for **B**, but with 12.8 nM *Sa*CoaBC. **D**) As for **B**, but with 25.5 nM *Sa*CoaBC. **E)** The fractional activity of *Sa*CoaBC tested at 25.5 nM (■) and 12.8 nM (●) as a function of [P-CJ]. The solid line represents the data fit to Morrison equation (Eqn. 2). **F)** Normalized heat-induced protein melting curves for *Sa*CoaBC determined by following the changes in the protein's secondary structure by circular dichroism (CD) spectroscopy. The four curves represent from left to right: the apo protein (●); protein with MgCTP (●); protein with MgCTP and P-Pan (**5**) (▼); and protein with MgCTP and P-CJ (**10**) (▲).

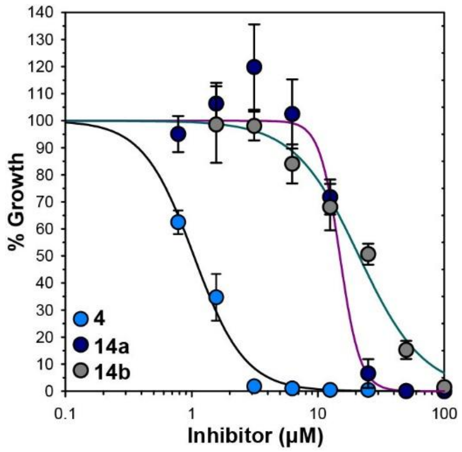

Supplementary Figure S2. Representative dose response curves of CJ-15,801 (4) and masked P-CJ prodrugs 14a and 14b inhibiting *S. aureus* growth. The growth of *S. aureus* Xen29 in tryptic soy broth (TSB) in the presence of the indicated compounds was assessed after 24h, and normalized to negative (uninoculated media) and positive (no inhibitor) controls.

MATERIALS & METHODS

General materials and methods. Unless otherwise stated, all chemicals and reagents were from Merck-Millipore/Sigma-Aldrich, Acros, Alfa Aesar, BeanTown Chemical, Fischer Scientific, TCI, or VWR and used without further purification. Anhydrous solvents were dried by MBRAUN MB-SPS solvent purification system before use. All reactions without aqueous reagents were carried out under nitrogen. All microwave assisted reactions were performed in a SmithSynthesizer (Biotage AB). Reaction temperatures were determined using the built-in, on-line IR-sensor. In the general procedure detailed below, the reaction vessel was not purged or put under an inert atmosphere. All NMR analyses were performed using 300 MHz Varian VNMRS, 400 MHz Varian Unity Inova, or 600 MHz Varian Unity Inova instruments at Stellenbosch University Central Analytical Facility (CAF) or on an Agilent spectrometer at 400 MHz (^{1}H) or 101 MHz (^{13}C) at George Washington University. Chemical shifts (δ) are given in ppm, with the abbreviations indicating multiplicity (s = singlet, t = triplet, q = quartet, m = multiplet), coupling constant (*J*) in Hz, and integration of the observed signals. Mass spectra were obtained in the ESI mode on an LC-MS (Agilent 1100 or Shimadzu 2020) or in the EI mode on a GC-MS (Shimadzu GCMS-QP2010S). High-resolution mass spectroscopy (HRMS) spectra were recorded in positive ESI mode on a Waters Q-TOF Ultima mass spectrometer (UIUC Mass Spectrometry Laboratory or Stellenbosch University CAF). Thin layer chromatography (TLC) was performed using Baker-flex Silica Gel IB2-F silica plates and column chromatography was carried out using silica gel (40-63 µm). The purity of synthesized compounds (>95%) was determined by $^{1}H/^{13}C$ NMR, LC-PDA-MS, and/or HRMS.

Synthesis of known compounds. CJ-15,801 (**4**) and P-CJ (**10**) were synthesised as described.[1] 5′-Azido-5′-deoxycytidine (**19**) was synthesized according to literature protocols with the use of sodium azide instead of lithium azide.[2] Bis(POM)-phosphoryl chloride (**27**) was prepared according to literature procedures.[3]

General procedure for microwave assisted nucleophilic ring-opening of D-(-)-pantolactone. D-(-)-Pantolactone (**20**) (300 mg, 2.31 mmol), Et_3N (482 µL, 3.46 mmol), and the appropriate propargylic amine (**16a–c**) were dissolved in EtOH (2 mL). The reaction vessel was sealed and subjected to microwave reaction conditions with mechanical stirring. Specific temperature data for nucleophilic ring opening of D-(-)-pantolactone in EtOH was: 120 s ramp time (ambient temperature to 160°C) and 1800 s hold time at 160°C. After air cooling, the reaction mixture was vented and the solvent removed under reduced pressure, before being purified by flash chromatography (EtOAc/hexane to MeOH/EtOAc) to

yield the products (**21a–c**) as single diastereomers.

(*R*)-Prop-2-yn-1-yl-3-(2,4-dihydroxy-3,3-dimethylbutanamido)-propanoate (17a).

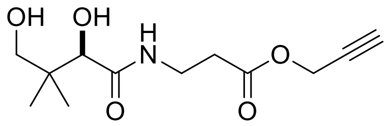

Calcium D-pantothenate (1.00 g, 2.11 mmol) was dissolved in anhydrous DMF (15 mL). Propargyl bromide (**15**, 80% in toluene (1.5 mL, 14 mmol) was added and the mixture was heated under reflux overnight at 80–90°C. The DMF was removed by lyophilization, and the product was purified by flash chromatography (EtOAc/hexane, 4:1) to yield a light brown clear oil (969 mg, 89%). ^{1}H NMR (300 MHz, $CDCl_3$) δ 7.17 (b. s, 1H), 4.70 (d, *J* = 2.4 Hz, 2H), 4.01 (d, *J* = 4.2 Hz, 1H), 3.65–3.54 (m, 3H), 3.50 (t, *J* = 4.1 Hz, 2H), 3.04–2.97 (m, 1H), 2.63 (t, *J* = 6.0 Hz, 2H), 2.49 (t, *J* = 2.5 Hz, 1H), 1.02 (s, 3H), 0.91 (s, 3H). ^{13}C NMR (151 MHz, $CDCl_3$) δ 173.28, 171.63, 77.77, 77.46, 75.32, 71.41, 52.36, 39.43, 34.65, 34.03, 21.44, 20.38.

(*R*)-2,4-Dihydroxy-3,3-dimethyl-*N*-[3-oxo-3-(prop-2-yn-1-ylamino)propyl]-butanamide (17b).

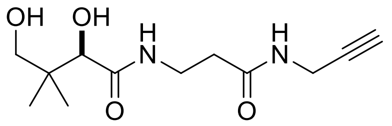

Sodium D-pantothenate (370 mg, 1.53 mmol) was dissolved in dry DMF (4.2 mL). DPPA (0.4 mL, 1.8 mmol) and propargylamine (**16a**, 0.12 mL, 1.84 mmol) were then added sequentially while stirring on ice. Finally, triethylamine (0.18 mL, 1.84 mmol) was added, and reaction stirred for 2 hours on ice, before allowing to warm to room temperature and stirring overnight. The solvent was removed by lyophilization and the solid then redissolved/resuspended in distilled water. Amberlite IRA-400 (OH^-) was added to this suspension until pH 12 was reached, after which the resin was removed by filtration and washed with water and then acetonitrile. The solvent was again removed by freeze-drying before purifying the resultant residue by column chromatography (CH_2Cl_2/MeOH, 9:1). **17b** was obtained as a clear light-yellow oil (184 mg, 47%). ^{1}H NMR (400 MHz, D_2O) δ 4.01–3.93 (m, 3H), 3.59–3.36 (m, 4H), 2.61 (t, *J* = 2.5 Hz, 1H), 2.52 (t, *J* = 6.4 Hz, 2H), 0.93 (s, 3H), 0.89 (s, 3H). ^{13}C NMR (151 MHz, D_2O) δ 175.75, 174.35, 130.44, 76.39, 69.01, 49.50 (MeOH), 39.20, 35.94, 35.75, 30.87, 29.39, 21.14, 19.73.

(*R*)-Prop-2-yn-1-yl 3-(4-{[bis(benzyloxy)phosphoryl]oxy}-2-hydroxy-3,3-dimethylbutanamido)-propanoate (18a).

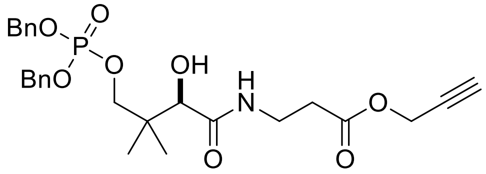

17a (260 mg, 1.01 mmol) was dissolved in dry DMF (2 mL). HATU (576 mg, 1.52 mmol) and dibenzylphosphate (295 mg, 1.06 mmol) were stirred together in dry DMF (5 mL) for 5 min until fully dissolved. The clear solution was then added to **23a**

and stirred for 10 min. Lastly, DIPEA (0.528 mL, 3.03 mmol) was added, and the reaction stirred at room temperature overnight. Upon addition of DIPEA the solution cycled through several different colours before stabilising to a reddish brown. The solvent was removed by lyophilization and the product purified by column chromatography (EtOAc/hexanes, 3:1) to give a pinkish gel/grease (158 mg, 30%) ^{1}H NMR (400 MHz, $CDCl_3$) δ 7.41–7.28 (m, 10H), 5.10–4.97 (m, 4H), 4.68 (s, 2H), 4.43 (d, *J* = 4.9 Hz, 1H), 4.05 (t, *J* = 9.1 Hz, 1H), 3.90 (d, *J* = 3.5 Hz, 1H), 3.59–3.46 (m, 3H), 2.60 (t, *J* = 6.4 Hz, 2H), 2.47 (t, *J* = 2.4 Hz, 1H), 1.07 (s, 3H), 0.78 (s, 3H). ^{13}C NMR (151 MHz, $CDCl_3$) δ 172.18, 171.41, 128.90, 128.88, 128.81, 128.16, 128.10, 77.56, 75.22, 73.88, 73.34, 69.99, 69.96, 69.94, 69.90, 52.25, 39.70, 39.67, 34.63, 34.08, 21.11, 18.43.

(*R*)-Dibenzyl (3-hydroxy-2,2-dimethyl-4-oxo-4-{[3-oxo-3-(prop-2-yn-1-ylamino)propyl]amino}butyl) phosphate (18b).

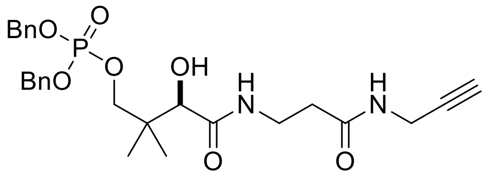

Same method followed as for **18a** above with the following changes. **17b** (467 mg, 1.83 mmol) was dissolved in dry DMF (5 mL). HATU (1040 mg, 2.74 mmol) and dibenzylphosphate (534 mg, 1.92 mmol) were stirred together in dry DMF (5 mL) for 5 min until fully dissolved. The clear solution was then added to **23b** and stirred for 10 min. Lastly, DIPEA (955 µL, 5.49 mmol) was added, and the reaction stirred at room temperature together for 72 hours. The solvent was removed by freeze-drying then redissolved in CH_2Cl_2 (100 mL) and washed with distilled water (6×40 mL). The organic layer was dried over magnesium sulfate, filtered and solvent removed by rotary evaporation. The crude oil was then purified with column chromatography (CH_2Cl_2/MeOH, 9:1) to give a yellow oil (20 mg, 2%). ^{1}H NMR (400 MHz, DMSO) δ 8.33 (, *J* = 5.5 Hz, 1H), 7.77 (app. t, *J* = 5.5 Hz, 1H), 7.44–7.31 (m, 10H), 5.68 (d, *J* = 5.5 Hz, 1H), 5.02 (d, *J* = 8.0 Hz, 4H), 3.95–3.77 (m, 4H), 3.67 (d, *J* = 5.6 Hz, 2H), 3.35–3.20 (m, 2H), 3.08 (t, *J* = 2.4 Hz, 1H), 2.69 (s, 1H), 2.29 (t, *J* = 7.2 Hz, 2H), 1.26 (m, 2H), 0.86 (s, 3H), 0.82 (s, 3H). The compound was used immediately without further characterization.

(*R*)-2,4-Dihydroxy-3,3-dimethyl-*N*-(prop-2-yn-1-yl)butanamide (21a).

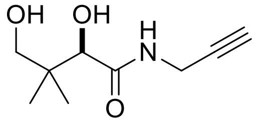

Propargylamine (**16a**, 222 µL, 3.46 mmol) was subjected to microwave reaction conditions in EtOH as detailed above, using D-(-)-pantolactone (**20**) as the limiting reagent. The reaction mixture was purified by flash chromatography (EtOAc/hexane, 1:1 to EtOAc/MeOH, 9:1) to yield an orange oil (272 mg, 64%). ^{1}H NMR (300 MHz, $CDCl_3$) δ 7.17 (t, *J* = 5.0 Hz, 1H), 4.42 (s, 1H), 4.05 (dd, *J* = 5.3, 2.7 Hz, 3H), 3.50 (s, 2H), 2.24 (t, *J* =

2.5 Hz, 1H), 1.00 (s, 3H), 0.92 (s, 3H). ^{13}C NMR (75 MHz, $CDCl_3$) δ 173.20, 79.21, 77.04, 71.60, 71.20, 39.40, 28.69, 20.99, 20.35. This data is in agreement with that reported previously.[4]

(*R*)-*N*-(But-3-yn-1-yl)-2,4-dihydroxy-3,3-dimethylbutanamide (21b).

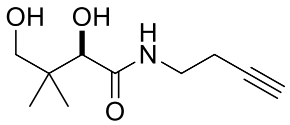

1-Amino-3-butyne (**16b**, 283 µL, 3.46 mmol) was subjected to microwave reaction conditions in EtOH as detailed above, using D-(-)-pantolactone (**20**) as the limiting reagent. The reaction mixture was purified by flash chromatography (EtOAc/hexane, 1:1 to EtOAc/MeOH, 19:1) to yield a yellow oil (296 mg, 64%). ^{1}H NMR (600 MHz, D_2O) δ 3.89 (s, 1H), 3.41 (d, J = 11.2 Hz, 1H), 3.36–3.23 (m, 3H), 2.33 (t, J = 6.6 Hz, 2H), 0.84 (s, 3H), 0.81 (s, 3H). ^{13}C NMR (151 MHz, D_2O) δ 175.13, 82.57, 75.85, 70.51, 68.38, 61.67, 38.56, 37.50, 20.46, 19.14, 18.47, 18.44, 13.22.

(*R*)-2,4-Dihydroxy-3,3-dimethyl-*N*-(pent-4-yn-1-yl)butanamide (21c).

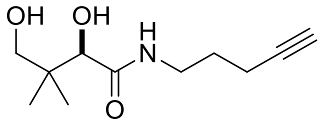

4-Pentyn-1-amine (**16c**, 335 µL, 3.46 mmol) was subjected to microwave reaction conditions in EtOH as detailed above, using D-(-)-pantolactone (**20**) as the limiting reagent. The reaction mixture was purified by flash chromatography (EtOAc/hexane, 1:1 to EtOAc/MeOH, 19:1) to yield a clear oil (380 mg, 77%). ^{1}H NMR (600 MHz, $CDCl_3$) δ 7.09 (t, J = 5.4 Hz, 1H), 4.45 (s, 1H), 4.00 (s, 1H), 3.91 (s, 1H), 3.47 (s, 2H), 3.45–3.31 (m, 2H), 2.24 (td, J = 7.0, 2.6 Hz, 2H), 1.99 (t, J = 2.6 Hz, 1H), 1.74 (apparent p, J = 6.9 Hz, 2H), 0.98 (s, 3H), 0.90 (s, 3H). ^{13}C NMR (75 MHz, $CDCl_3$) δ 173.20, 79.21, 77.04, 71.60, 71.20, 39.40, 28.69, 20.99, 20.35.

General procedure for the copper-catalyzed azide-alkyne cycloadditions. The general procedure for the copper-catalyzed synthesis of 1,4-disubstituted 1,2,3-triazoles is described for **12c**. 5′-Azido-5′-deoxycytidine[2] (**19**) (35 mg, 130 µmol, 1.2 equiv.) and **21a** (20 mg, 108 µmol) were suspended in a 1:1:2:1 mixture of water/*tert*-butyl alcohol/acetonitrile/DMF mixture (1.2 mL). Sodium ascorbate (130 µL of freshly prepared 1M solution in water, 130 µmol, 1.2 equiv.) was added, followed by copper (II) sulfate pentahydrate (3.3 mg in 100 µL of water, 13 µmol, 0.12 equiv.). The cloudy mixture was stirred vigorously overnight, at which point it became clear and TLC analysis indicated complete consumption of the reactants. The reaction mixture was reduced *in vacuo* and purified via silica chromatography to afford the pure product.

(*R*)-*N*-{[1-({[2*R*,3*S*,4*R*,5*R*]-5-[4-amino-2-oxopyrimidin-1(2*H*)-yl]-3,4-dihydroxytetrahydrofuran-2-yl}methyl)-1*H*-1,2,3-triazol-4-yl]methyl}-2,4-dihydroxy-3,3-dimethylbutanamide (12c).

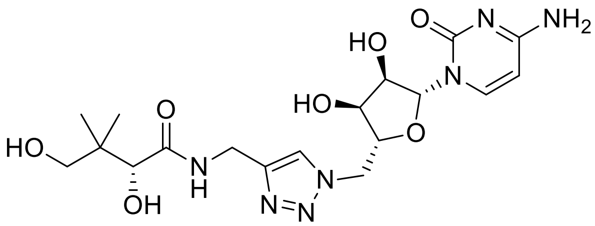

21a (20.0 mg, 108 µmol), as the limiting reagent, was subjected to reaction conditions as detailed above. The reaction mixture was purified by flash chromatography (EtOAc/hexane, 4:1 to EtOAc/MeOH/acetonitrile/H_2O, 5:1:1:1) to yield a clear oil (49 mg, 83%). ^{1}H NMR (300 MHz, CD_3OD) δ 7.92 (s, 1H), 7.52 (d, *J* = 7.5 Hz, 1H), 5.97 (d, *J* = 7.5 Hz, 1H), 5.72 (d, *J* = 3.5 Hz, 1H), 4.84–4.69 (m, 1H), 4.51 (s, 1H), 4.25 (ddd, *J* = 9.1, 6.0, 3.7 Hz, 1H), 4.16–4.07 (m, 1H), 4.00 (s, 1H), 3.51–3.34 (m, 1H), 3.32 (dt, *J* = 3.3, 1.6 Hz, 1H), 0.94 (s, 1H), 0.90 (s, 1H). ^{13}C NMR (75 MHz, CD_3OD) δ 176.30, 167.39, 158.14, 146.21, 143.83, 125.69, 96.75, 94.35, 82.73, 77.50, 74.66, 71.79, 70.14, 52.42, 49.00, 40.27, 35.10, 21.60, 21.43.

(*R*)-*N*-{2-[1-({[2*R*,3*S*,4*R*,5*R*]-5-[4-amino-2-oxopyrimidin-1(2*H*)-yl]-3,4-dihydroxytetrahydrofuran-2-yl}methyl)-1*H*-1,2,3-triazol-4-yl]ethyl}-2,4-dihydroxy-3,3-dimethylbutanamide (12d).

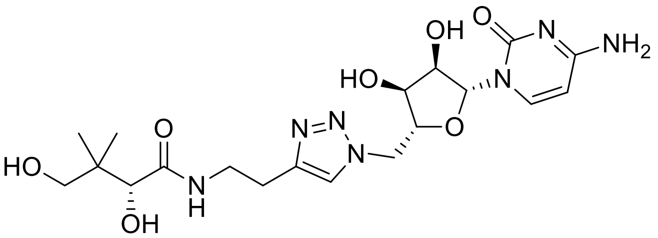

21b (22.0 mg, 108 µmol), as the limiting reagent, was subjected to reaction conditions as detailed above. The reaction mixture was purified by flash chromatography (EtOAc/hexane, 4:1 to EtOAc/MeOH/acetonitrile/H_2O, 5:1:1:1) to yield a clear oil (70 mg, 99%). ^{1}H NMR (600 MHz, CD_3OD) δ ^{1}H NMR (600 MHz, CD_3OD) δ 7.87 (s, 1H), 7.46 (d, *J* = 7.5 Hz, 1H), 5.94 (d, *J* = 7.2 Hz, 1H), 5.75 (d, *J* = 3.5 Hz, 1H), 4.81 (dd, *J* = 14.6, 3.4 Hz, 1H), 4.73 (dd, *J* = 14.6, 7.0 Hz, 1H), 4.60 (s, 1H), 4.27 (td, *J* = 6.8, 3.4 Hz, 1H), 4.22 (dd, *J* = 5.6, 3.6 Hz, 1H), 4.10 (t, *J* = 6.1 Hz, 1H), 3.92 (s, 1H), 3.56 (t, *J* = 6.9 Hz, 1H), 3.44 (d, *J* = 11.0 Hz, 1H), 3.36 (d, *J* = 11.1 Hz, 1H), 2.95 (t, *J* = 6.9 Hz, 2 H), 0.91 (s, 3H), 0.88 (s, 3H). ^{13}C NMR (151 MHz, CD_3OD) δ 176.15, 167.51, 158.14, 146.38, 143.62, 132.40, 129.85, 125.22, 96.53, 94.32, 82.80, 77.58, 74.73, 72.06, 70.30, 70.22, 40.21, 39.46, 26.47, 21.45, 21.15.

(*R*)-*N*-{3-[1-({[2*R*,3*S*,4*R*,5*R*]-5-[4-amino-2-oxopyrimidin-1(2*H*)-yl]-3,4-dihydroxytetrahydrofuran-2-yl}methyl)-1*H*-1,2,3-triazol-4-yl]propyl}-2,4-dihydroxy-3,3-dimethylbutanamide (12e).

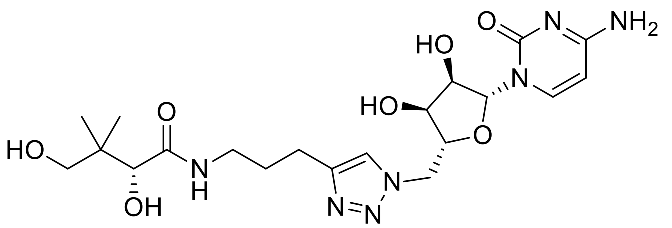

21c (23.0 mg, 108 µmol), as the limiting reagent, was subjected to reaction conditions as detailed above. The reaction mixture was purified by flash chromatography (EtOAc/hexane, 4:1 to EtOAc/MeOH/acetonitrile/H_2O, 5:1:1:1) to yield a clear oil (63 mg, 96%). ^{1}H NMR (300 MHz, CD_3OD) δ 7.83 (s, 1H), 7.39 (d, *J* = 7.5 Hz, 1H), 5.91 (d, *J* = 7.4 Hz, 1H), 5.76 (d, *J* = 3.4 Hz, 1H), 4.84–4.66 (m, 3H), 4.27 (td, *J* = 6.5, 3.6 Hz, 1H), 4.16 (dd, *J* = 5.6, 3.4 Hz, 1H), 4.12–4.04 (m, 1H), 3.94 (s, 1H), 3.52–3.36 (m, 2H), 3.28 (t, *J* = 7.0 Hz, 2H), 2.75 (t, *J* = 7.5 Hz, 2H), 1.95–1.83 (m, 2H), 0.95 (s, 2H), 0.94 (s, 2H). ^{13}C NMR (75 MHz, CD_3OD) δ 176.07, 167.38, 158.02, 148.42, 143.38, 124.84, 93.98, 82.75, 77.65, 74.82, 71.99, 70.34, 52.32, 49.00, 40.27, 39.24, 30.22, 23.63, 21.50, 21.17.

[1-({[2*R*,3*S*,4*R*,5*R*]-5-[4-amino-2-oxopyrimidin-1(2*H*)-yl]-3,4-dihydroxytetrahydrofuran-2-yl}meth-yl)-1*H*-1,2,3-triazol-4-yl]methyl 3-[(*R*)-2,4-dihydroxy-3,3-dimethylbutanamido]propanoate (12a).

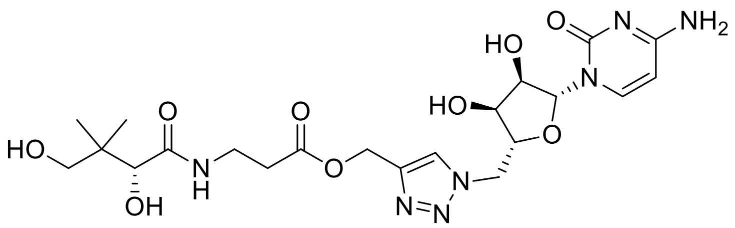

17a (28.0 mg, 108 µmol), as the limiting reagent, was subjected to reaction conditions as detailed above. The reaction mixture was purified by flash chromatography (EtOAc/hexane, 4:1 to EtOAc/MeOH/acetonitrile/H_2O, 5:1:1:1)) to yield a clear oil (53 mg, 93%). ^{1}H NMR (300 MHz, CD_3OD) δ 8.07 (s, 1H), 7.40 (d, *J* = 7.5 Hz, 1H), 5.79 (d, *J* = 3.3 Hz, 1H), 5.23 (s, 2H), 4.94 (s, 1H), 4.86–4.72 (m, 3H), 4.61 (s, 2H), 4.29 (td, *J* = 6.6, 3.5 Hz, 2H), 4.20–4.08 (m, 2H), 3.92 (s, 1H), 3.51 (t, *J* = 6.7 Hz, 2H), 3.47–3.34 (m, 3H), 3.33 (dt, *J* = 3.3, 1.6 Hz, 3H), 2.61 (t, *J* = 6.6 Hz, 2H), 0.92 (s, 3H), 0.90 (s, 3H). ^{13}C NMR (75 MHz, CD_3OD) δ 176.13, 173.03, 143.25, 127.21, 93.83, 82.74, 77.50, 74.82, 72.04, 70.24, 58.39, 52.56, 49.00, 40.23, 35.84, 34.77, 21.42, 21.14. HRMS (ESI$^+$-TOF) *m/z* $[M+H]^+$ calcd for $C_{21}H_{32}N_7O_9$ 526.2262; found: 526.2265.

(*R*)-*N*-[3-({[1-({[2*R*,3*S*,4*R*,5*R*]-5-[4-amino-2-oxopyrimidin-1(2*H*)-yl]-3,4-dihydroxytetrahydrofuran-2-yl}methyl)-1*H*-1,2,3-triazol-4-yl]methyl}amino)-3-oxopropyl]-2,4-dihydroxy-3,3-dimethylbutan-amide (12b).

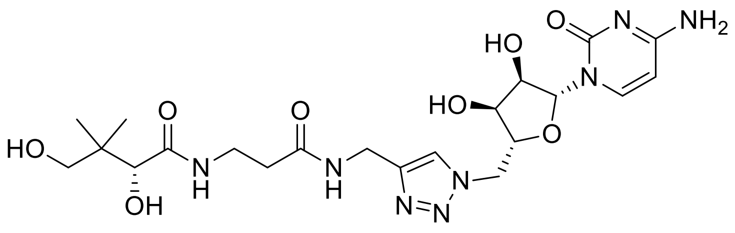

17b (28.0 mg, 108 µmol), as the limiting reagent, was subjected to reaction conditions as detailed above. The reaction mixture was purified by flash chromatography (EtOAc/hexane, 4:1 to EtOAc/MeOH/acetonitrile/H_2O, 5:1:1:1)) to yield a clear oil (47 mg, 93%). ^{1}H NMR (400 MHz, CD_3OD) δ 7.91 (s, 1H), 7.44 (d, *J* = 7.5 Hz, 1H), 5.95 (s, 1H), 5.73 (d, *J* = 3.0 Hz, 1H), 4.81–4.69 (m, 4H), 4.43 (s, 2H), 4.26 (td, *J* = 6.5, 3.5 Hz, 1H), 4.19 (dd, *J* = 10.5, 5.0 Hz, 1H), 4.09 (t, *J* = 6.0 Hz, 1H), 3.91 (s, 1H), 3.50 (t, *J* = 6.5 Hz, 2H), 3.48–3.36 (m, 2H), 3.34 (s, 1H), 3.30 (dt, *J* = 3.3, 1.6 Hz, 2H), 2.47 (t, *J* = 6.5 Hz, 2H), 0.89 (d, *J* = 2.7 Hz, 6H). ^{13}C NMR (101 MHz, CD_3OD) δ 176.12, 173.81, 167.45, 158.23, 143.55, 125.65, 94.05, 82.78, 77.43, 74.71, 71.93, 70.16, 52.53, 49.00, 40.28, 36.41, 36.27, 35.58, 21.34, 21.26. HRMS (ESI$^+$-TOF) *m*/*z* $[M+H]^+$ calcd for $C_{21}H_{33}N_8O_8$ 525.2421; found: 525.2417.

[1-({[2*R*,3*S*,4*R*,5*R*]-5-[4-amino-2-oxopyrimidin-1(2*H*)-yl]-3,4-dihydroxytetrahydrofuran-2-yl}meth-yl)-1*H*-1,2,3-triazol-4-yl]methyl 3-({*R*}-4-{[bis(benzyloxy)phosphoryl]oxy}-2-hydroxy-3,3-dimethyl-butanamido)propanoate (22a).

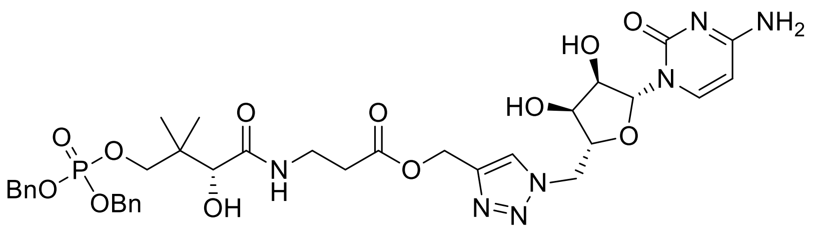

As per the general method with the following exceptions. **18a** (23.6 mg, 0.0456 mmol), **19**[2] (36.7 mg, 0.137 mmol), copper (II) sulfate pentahydrate (1.40 mg, 5.47 µmol), sodium-L-ascorbate (10.8 mg, 54.7 µmol) stirred at room temperature for 48 h. Diluted with distilled water and washed three times with CH_2Cl_2. The organic layers were combined, dried over magnesium sulfate and filtered. The solvent was removed under reduced pressure and the crude oil purified by column chromatography (EtOAc/MeOH/acetonitrile/H_2O, 5:1:1:1) to obtain a clear oil (18.1 mg, 50.6%). ^{1}H NMR (600 MHz, DMSO) δ 8.10 (s, 1H), 7.86 (s, 1H), 7.47–7.27 (m, 10H), 7.26–7.10 (m, 2H), 5.78–5.68 (m, 3H), 5.41 (s, 1H), 5.31 (s, 1H), 5.15–5.07 (m 2H), 5.06–4.97 (m 4H), 4.75–4.59 (m, 2H), 4.16–3.65 (m, 6H), 3.39–3.23 (m, 2H), 0.84 (s, 3H), 0.81 (s, 3H). The compound was used immediately without further characterization.

(*R*)-4-{[3-({[1-({[2*R*,3*S*,4*R*,5*R*]-5-[4-amino-2-oxopyrimidin-1(2*H*)-yl]-3,4-dihydroxytetrahydrofuran-2-yl}methyl)-1*H*-1,2,3-triazol-4-yl]methyl}amino)-3-oxopropyl]amino}-3-hydroxy-2,2-dimethyl-4-oxobutyl dibenzyl phosphate (22b).

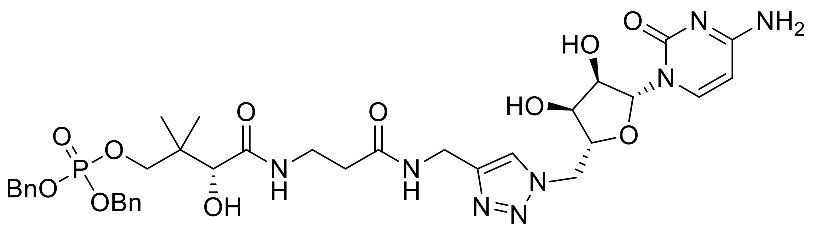

As per the general method with the following exceptions. **18b** (20.0 mg, 38.7 µmol), **19**[2] (31.2 mg, 116 µmol), copper (II) sulfate pentahydrate (1.2 mg, 4.6 µmol) and sodium ascorbate (9.2 mg, 46 µmol) were stirred together for 24 hours and then heated at 50°C for 24 hours before the solvent was removed by lyophilization. Additional copper (II) sulfate (10%) was added after 3 hours of heating with 10 additional equivalents of sodium ascorbate. A further 10 equivalents sodium ascorbate were added at 7 hours after heating. The resultant crude yellow oil was purified by column chromatography (CH_2Cl_2/MeOH, 9:1) resulting in a clear oil (4.5 mg, 14%). ^{1}H NMR (600 MHz, DMSO) δ 8.40 (app. t, *J* = 5.5 Hz, 1H), 7.95 (s, 1H), 7.82 (app. t, *J* = 5.5 Hz, 1H), 7.78–7.73 (m, 1H), 7.41–7.33 (m, 10H), 7.28–7.11 (m, 2H), 5.78–5.70 (m, 3H), 5.02 (d, *J* = 7.4 Hz, 4H), 4.70–4.55 (m, 2H), 4.31–3.75 (m, 7H), 2.29 (t, *J* = 7.2 Hz, 2H), 0.84 (s, 3H), 0.81 (s, 3H). The compound was used immediately without further characterization.

[1-({[2*R*,3*S*,4*R*,5*R*]-5-[4-amino-2-oxopyrimidin-1(2*H*)-yl]-3,4-dihydroxytetrahydrofuran-2-yl}methyl)-1*H*-1,2,3-triazol-4-yl]methyl 3-[(*R*)-2-hydroxy-3,3-dimethyl-4-(phosphonooxy)-butanamido]propanoate (13a).

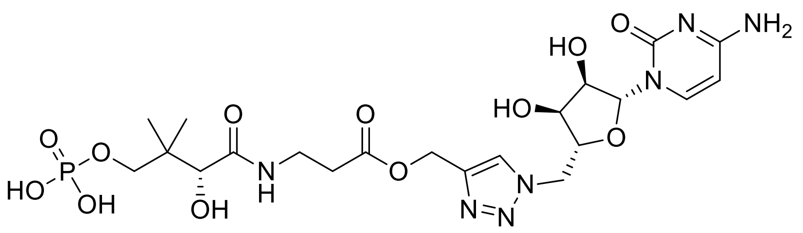

22a (18.1 mg, 23.0 µmol) was dissolved in 10% distilled water/MeOH. Pd/C (10%) (3.68 mg, 34.5 µmol) was added and the reaction stirred under an atmosphere of hydrogen (balloon) for 8h. Suspension filtered off and solvent removed to obtain the final product as a clear oil (11.9 mg, 85%). ^{1}H NMR (400 MHz, D_2O) δ 8.15 - 8.09 (m, 1H), 7.34–7.28 (m, 1H), 6.26–5.97 (b. s, 1H), 5.84–5.76 (m, 1H), 5.30–5.21 (m, 2H), 4.98–4.88 (m, 1H), 4.46–4.25 (m, 2H), 4.17–3.99 (m, 3H), 3.83–3.72 (m, 1H), 3.59–3.46 (m, 3H), 3.35–3.12 (m, 1H), 2.74–2.63 (m, 3H), 0.89 (s, 3H), 0.79 (s, 3H). HRMS (ESI$^+$-TOF) *m*/*z* [M+H]$^+$ calcd for $C_{21}H_{33}N_7O_{12}P$ 606.1925; found: 606.1926.

(*R*)-4-{[3-({[1-({[2*R*,3*S*,4*R*,5*R*]-5-[4-amino-2-oxopyrimidin-1(2*H*)-yl]-3,4-dihydroxytetrahydrofuran-2-yl}methyl)-1*H*-1,2,3-triazol-4-yl]methyl}amino)-3-oxopropyl]amino}-3-hydroxy-2,2-dimethyl-4-oxobutyl dihydrogen phosphate (13b).

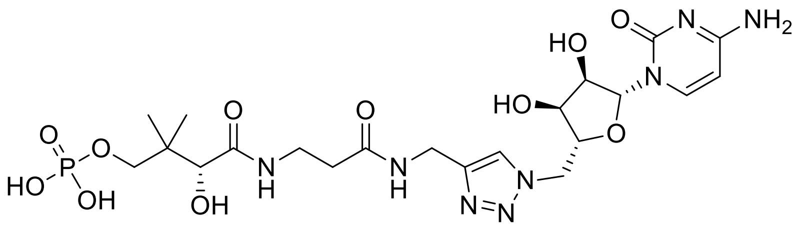

Same procedure as above with **22b** (4.5 mg, 5.7 μmol) and Pd/C (10%) (0.92 mg, 8.6 μmol) stirred for 18h. Clear oil obtained (2.5 mg, 73%). ^{1}H NMR (600 MHz, D_2O) δ 8.46 (s, 1H), 7.94 (s, 1H), 7.20–7.16 (m, 1H), 6.01–5.95 (m, 1H), 5.82–5.78 (m, 1H), 4.51–4.33 (m, 3H), 4.27–4.19 (m, 1H), 4.15–4.05 (m, 3H), 3.81–3.62 (m, 5H), 3.58–3.46 (m, 3H), 3.40–3.34 (m, 1H), 2.56–2.47 (m, 3H), 0.92 (s, 3H), 0.75 (s, 3H).

[(2,2-Dimethylpropanoyl)oxy]methyl (2*E*)-3-bromoprop-2-enoate (23a).

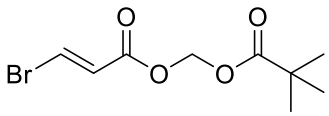

To a solution of (2E)-3-bromoprop-2-enoic acid5 (1.51 g, 10.0 mmol) in DMF (15 mL) was added triethylamine (2.63 mL, 18.9 mmol), chloromethyl-pivalate (2.87 mL, 19.9 mmol), and sodium iodide (100 mg, 0.7 mmol). The reaction was allowed to stir at 70oC for 15 minutes and was then quenched with water. The mixture was extracted with diethyl ether (3×20 mL), dried over sodium sulfate, filtered, and evaporated. The residue was purified by normal phase column chromatography using hexanes/EtOAc, 30:1 to yield a colorless oil (416 mg, 16%). ^{1}H NMR (400 MHz, $CDCl_3$) δ 7.71 (d, *J* = 13.9 Hz, 1H), 6.55 (d, *J* = 13.9 Hz, 1H), 5.82 (s, 2H), 1.22 (s, 9H). ^{13}C NMR (101 MHz, $CDCl_3$) δ 177.11, 162.51, 128.95, 127.72, 79.62, 26.84.

(5-Methyl-2-oxo-2H-1,3-dioxol-4-yl)methyl (2*E*)-3-bromoprop-2-enoate (23b).

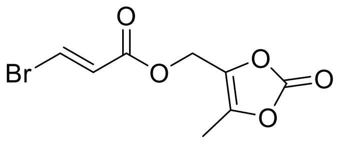

Sodium carbonate (0.49 g, 4.6 mmol) and 4-chloromethyl-5-methyl-1,3-dioxone-2-one (0.98 g, 6.6 mmol) were weighed and added to a round bottom flask. (2*E*)-3-bromoprop-2-enoic acid[5] (1.50 g, 9.9 mmol) in DMF (15 mL) was added to the flask, and the reaction mixture was stirred overnight at room temperature. The color changed to milky yellow. The reaction mixture was extracted between EtOAc and water. The organic portion was washed with brine, dried over $MgSO_4$, filtered, and evaporated under reduced pressure. The crude oil was purified by column chromatography (hexanes/ EtOAc, 5:1) to yield the desired product (0.89 g, 54%). ^{1}H NMR (400 MHz, $CDCl_3$) δ 7.69 (d, *J* = 13.9 Hz, 1H), 6.54 (d, *J* = 13.9 Hz, 1H), 4.92 (s, 2H), 2.19 (s, 3H). The compound was used immediately without further

characterization.

[3-(Trifluoromethyl)phenyl]methyl (2*E*)-3-bromoprop-2-enoate (23c).

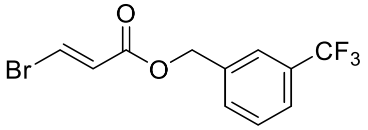

To *N*,*N*'-dicyclohexylcarbodiimide (1.025 g, 4.968 mmol), and (2*E*)-3-bromoprop-2-enoic acid[5] (1.50 g, 9.94 mmol) in CH_2Cl_2 (10mL) at 0 °C, a solution of DMAP (0.016 g, 0.13 mmol) in CH_2Cl_2 (1 mL) was added. 3-Hydroxymethylbenzyl trifluoride (0.79 g, 4.5 mmol) was added dropwise. The reaction was allowed to stir at 0°C under a nitrogen atmosphere for 1 hr, and at room temperature overnight. The cloudy reaction was filtered and extracted with 2 M $NaHCO_3$ and then 2M HCl. The organic solvent was dried over $MgSO_4$, filtered, and evaporated. The crude mixture was purified by column chromatography (CH_2Cl_2/hexanes, 1:1) to yield a clear oil (1.90 g, 70.1%). ^{1}H NMR (400 MHz, $CDCl_3$) δ 7.71–7.46 (m, 5H), 6.59 (d, *J* = 13.8 Hz, 1H), 5.23 (s, 2H). ^{13}C NMR (101 MHz, $CDCl_3$) δ 163.72, 136.42, 131.52, 129.19, 128.22, 127.78, 125.32, 125.28, 124.99, 65.82.

Prop-2-en-1-yl-(2*E*)-3-bromoprop-2-enoate (23d).

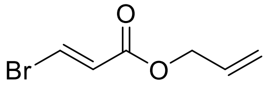

To a flask containing (2*E*)-3-bromoprop-2-enoic acid[5] (7.6 g, 50 mmol) in CH_2Cl_2 (100 mL) was added allyl alcohol (1.9 mL, 34 mmol) and DMAP (615.0 mg, 5.03 mmol). The solution was cooled to 0°C and diisopropylcarbodiimide (6.2 mL, 40 mmol) was added dropwise. The reaction was warmed to room temperature and stirred overnight. Product formation was monitored by thin-layer chromatography, after which the mixture was filtered, washed with saturated $NaHCO_3$ (3×100 mL) and brine (3×100 mL), and dried over $MgSO_4$. The crude mixture was concentrated *in vacuo* and purified by column chromatography (silica gel; CH_2Cl_2) to give the pure compound as a colorless oil (3.9 g, 61%). ^{1}H NMR (400 MHz, $CDCl_3$): δ 7.63 (d, *J* = 13.9 Hz, 1H), 6.55 (d, *J* = 13.9 Hz, 1H), 5.99–5.88 (m, 1H), 5.34 (dq, *J* = 17.2, 1.4 Hz, 1H), 5.27 (dq, *J* = 10.4, 1.3 Hz, 1H), 4.66 (dt, *J* = 5.8, 1.5 Hz, 2H). ^{13}C NMR (101 MHz, $CDCl_3$) δ 163.80, 131.77, 128.64, 127.17, 118.83, 65.66.

[(2,2-dimethylpropanoyl)oxy]methyl (2*E*)-3-{[(4*S*)-2,2,5,5-tetramethyl-1,3-dioxan-4-yl]formamido}prop-2-enoate (25a).

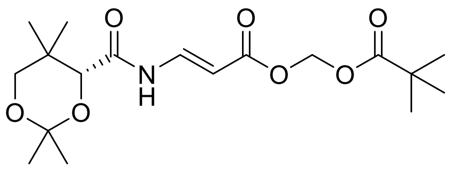

24[6] (256 mg, 1.37 mmol), Pd(OAc)$_2$ (34 mg, 0.15 mmol), Xantphos (131 mg, 0.226 mmol), K_2CO_3 (415 mg, 3.00 mmol), and CTAB (110 mg, 0.30 mmol) were combined in an oven-dried round bottomed flask. **23a** (400 mg, 1.51 mmol) in toluene (3.4 mL) was added to the flask. After stirring at 55°C for one hour, water (0.08 mL) was added to the

reaction mixture. The reaction was allowed to continue stirring for one hour at 55 °C. The reaction was diluted with EtOAc and washed between brine and EtOAc (3×5 mL). The organic layers were combined and dried over $MgSO_4$, filtered through Celite, and evaporated. The residue was purified via a silica plug using hexanes/ether, 3:1 to yield the desired product as an oil (350 mg, 69%). ^{1}H NMR (400 MHz, $CDCl_3$) δ 8.56 (d, *J* = 11.9 Hz, 1H), 7.96 (dd, *J* = 14.2, 11.9 Hz, 1H), 5.74 (s, 2H), 5.58 (d, *J* = 14.2 Hz, 1H), 4.15 (s, 1H), 3.65 (d, *J* = 11.9, 1H), 3.25 (d, *J* = 11.8 Hz, 1H), 1.43 (s, 3H), 1.39 (s, 3H), 1.34 (s, 9H), 0.98 (s, 3H), 0.95 (s, 3H). ^{13}C NMR (101 MHz, $CDCl_3$) δ 177.19, 168.14, 165.78, 101.45, 99.55, 79.32, 77.22, 71.19, 38.73, 33.40, 29.38, 26.85, 21.82, 18.84, 18.66.

(5-methyl-2-oxo-2*H*-1,3-dioxol-4-yl)methyl (2*E*)-3-{[(4*S*)-2,2,5,5-tetramethyl-1,3-dioxan-4-yl]formamido}prop-2-enoate (25b).

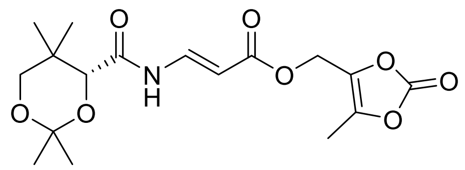

24[6] (411 mg, 2.20 mmol), $Pd(OAc)_2$ (45 mg, 0.20 mmol), Xantphos (174 mg, 0.300 mmol), K_2CO_3 (553 mg, 4.00 mmol), and CTAB (146 mg, 0.401 mmol) were combined in an oven-dried round bottomed flask. **23b** (500 mg, 2 mmol) in toluene (5 mL) was added to the flask. After stirring at 55°C for one hour, water (0.1 mL) was added to the reaction mixture. The reaction was allowed to continue stirring for one hour at 55°C. The reaction was diluted with EtOAc and washed between brine and EtOAc (3×5 mL). The organic layers were combined and dried over $MgSO_4$, filtered through Celite, and evaporated. The residue was purified via a silica plug using hexanes/ether, 2:1 to yield the desired product as a light brown solid (540 mg, 73%). ^{1}H NMR (400 MHz, $CDCl_3$) δ 8.46 (bs, 1H), 8.01 (dd, *J* = 14.2, 12.0 Hz, 1H), 5.61 (d, *J* = 14.2 Hz, 1H), 4.89 (s 2H), 4.20 (s, 1H), 3.72 (d, *J* = 12 Hz, 1H), 3.32 (d, *J* = 11.8 Hz, 1H), 2.18 (s 3H), 1.51 (s, 3H), 1.44 (s, 3H), 1.05 (s, 3H), 1.00 (s, 3H). ^{13}C NMR (101 MHz, $CDCl_3$) δ 171.28, 166.61, 152.31, 140.08, 137.61, 133.91, 101.49, 99.76, 77.39, 71.37, 53.59, 33.59, 29.58, 21.99, 18.98, 18.83, 9.53.

[3-(Trifluoromethyl)phenyl]methyl (2*E*)-3-{[(4*S*)-2,2,5,5-tetramethyl-1,3-dioxan-4-yl]formamido}-prop-2-enoate (25c).

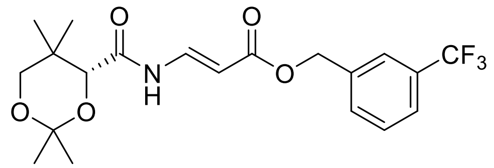

24[6] (333 mg, 1.78 mmol), $Pd(OAc)_2$ (36 mg, 0.16 mmol), Xantphos (140 mg, 0.24 mmol), K_2CO_3 (442 mg, 3.20 mmol), and CTAB (116 mg, 0.318 mmol) were combined in an oven-dried round bottomed flask. **23c** (500 mg, 1.62 mmol) in toluene (5 mL) was added to the flask. After stirring at 55°C for one hour, water (0.1 mL) was added to the reaction mixture. The reaction was allowed to continue stirring for one hour at 55°C. The reaction

was diluted with EtOAc, filtered through celite, and washed between brine and EtOAc (3×5 mL). The organic layers were combined and dried over $MgSO_4$, filtered, and evaporated. The residue was purified via a silica plug using hexanes/ether, 3:1 to yield the desired product as a light yellow oil (610 mg, 91%). ^{1}H NMR (400 MHz, $CDCl_3$) δ 8.43 (d, *J* = 12.0 Hz, 1H), 8.02 (dd, *J* = 14.1, 11.4 Hz, 1H), 7.65–7.43 (m, 4H), 5.68 (d, *J* = 14.2 Hz, 1H), 5.23 (s, 2H), 4.20 (s, 1H), 3.71 (d, *J* = 11.8, 1H), 3.32 (d, *J* = 11.8 Hz, 1H), 1.51 (s, 3H), 1.45 (s, 3H), 1.05 (s, 3H), 1.01 (s, 3H). ^{13}C NMR (101 MHz, $CDCl_3$) δ 168.16, 166.91, 137.43, 136.95, 131.45, 129.18, 125.09, 125.05, 124.88, 124.84, 102.39, 99.74, 77.39, 71.40, 65.19, 33.58, 29.58, 22.00, 18.98, 18.83.

Prop-2-en-1-yl-(2*E*)-3-{[((4*R*)-2,2,5,5-tetramethyl-1,3-dioxan-4-yl)carbonyl]amino}prop-2-enoate (25d).

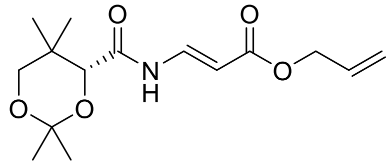

To an oven dried flask were added vinyl bromide **23d** (3.8 g, 20 mmol), amide **24**[6] (2.5 g, 13 mmol), $Pd(OAc)_2$ (298 mg, 1.33 mmol), Xantphos (1.2 g, 2.1 mmol), CTAB (967 mg, 2.65 mmol), and K_2CO_3 (3.7 g, 27 mmol) all dissolved in toluene (40 mL). The reaction was heated to 55°C for 1 hour, upon which water (0.7 mL, 40 mmol) was added and the mixture was stirred for a further 1 hour at 55°C, with product formation monitored by thin-layer chromatography. After cooling to room temperature, the solvent was removed *in vacuo*, after which the reaction was redissolved in EtOAc, washed with brine (2×100 mL), and dried over Na_2SO_4. The crude mixture was filtered and concentrated *in vacuo* by rotary evaporation. The desired compound was obtained after purification by column chromatography (silica gel; hexanes/EtOAc) as a yellow solid (1.7 g, 44%). ^{1}H NMR (400 MHz, $CDCl_3$): δ 8.40 (d, *J* = 12.3, 1H), 8.00 (dd, *J* = 14.2, 12.0 Hz, 1H), 5.97–5.87 (m, 1H), 5.61 (d, *J* = 14.2 Hz, 1H), 5.33 (dq, *J* = 17.2, 1.6 Hz, 1H), 5.21 (dq, *J* = 10.4, 1.4 Hz, 1H), 4.63 (dt, *J* = 5.68, 1.5 Hz, 2H), 4.18 (s, 1H), 3.69 (d, *J* = 12.6 Hz, 1H), 3.30 (d, *J* = 11.7 Hz 1H), 1.50 (s, 3H), 1.44 (s, 3H), 1.03 (s, 3H), 0.99 (s, 3H). MS (ESI-) *m/z* 295.95 $[M-H]^-$.

[(2,2-dimethylpropanoyl)oxy]methyl (2E)-3-[(2S)-2,4-dihydroxy-3,3-dimethylbutanamido]prop-2-enoate (26a).

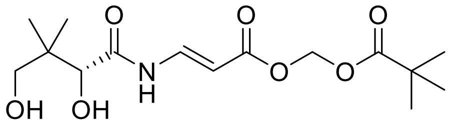

To a solution of **25a** (350 mg, 0.94 mmol) in CH_3CN (16.5 mL) was added $BiCl_3$ (104 mg, 0.330 mmol) and water (0.25 mL, 14 mmol). The reaction was allowed to stir overnight at room temperature. The reaction mixture was filtered, evaporated and diluted with EtOAc. The organic portion was washed with saturated $NaHCO_3$, (2×20 mL), dried over $MgSO_4$, filtered, and evaporated to give an

oily residue. The residue was purified using column chromatography with hexanes/EtOAc, 5:1 to give the desired product as a yellow solid (180 mg, 58%). ^{1}H NMR (400 MHz, $CDCl_3$) δ 8.97 (d, J = 12.0 Hz, 1H), 8.07 (dd, J = 14.2, 12.0 Hz, 1H), 5.81 (s, 2H), 5.59 (d, J = 14.2 Hz, 1H), 4.29 (bs, 1H), 4.21–4.18 (m, 1H), 3.64 (d, J = 10.9 Hz, 1H), 3.56 (d, J = 10.9 Hz, 1H), 2.50 (bs, 1H), 1.21 (s, 9H), 1.05 (s, 3H), 0.99 (s, 3H). ^{13}C NMR (101 MHz, $CDCl_3$) δ 177.55, 171.07, 166.01, 138.29, 101.40, 79.57, 78.54, 72.11, 39.45, 38.95, 27.03, 21.11, 20.26.

(5-methyl-2-oxo-2H-1,3-dioxol-4-yl)methyl (2E)-3-[(2S)-2,4-dihydroxy-3,3-dimethylbutanamido]-prop-2-enoate (26b).

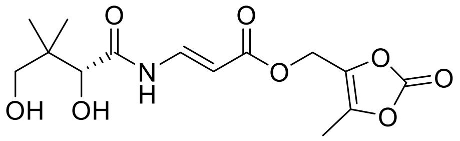

To a solution of **25b** (400 mg, 1 mmol) in CH_3CN (19 mL) was added $BiCl_3$ (120 mg, 0.38 mmol) and water (0.29 mL, 16 mmol). The reaction was allowed to stir overnight at room temperature. Aq. $NaHCO_3$ was added. The reaction mixture was extracted with EtOAc (3×5mL), dried over $MgSO_4$, filtered, and evaporated to give an oily residue. The residue was purified via a silica plug using hexanes/ether, 1:2 to give the desired product as a light yellow solid (251 mg, 70%). ^{1}H NMR (400 MHz, $CDCl_3$) δ 8.92 (d, J = 12.0 Hz, 1H), 8.05 (dd, J = 14.2, 12.0 Hz, 1H), 5.57 (d, J = 14.2 Hz, 1H), 4.90 (s, 2H), 4.20 (s, 1H), 3.65 (d, J = 10.8 Hz, 1H), 3.56 (d, J = 10.8 Hz, 1H), 2.19 (s, 3H), 1.05 (s, 3H), 0.99 (s, 3H). The compound was used immediately without further characterization.

[3-(trifluoromethyl)phenyl]methyl (2E)-3-[(2S)-2,4-dihydroxy-3,3-dimethylbutanamido]prop-2-enoate (26c).

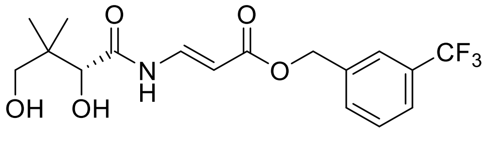

To a solution of **25c** (400 mg, 1 mmol) in CH_3CN (20 mL) was added $BiCl_3$ (107 mg, 0.34 mmol) and water (0.26 mL, 14 mmol). The reaction was allowed to stir overnight at room temperature. The reaction was filtered and washed with aqueous $NaHCO_3$. The reaction mixture was extracted with EtOAc (3×5mL), dried over $MgSO_4$, filtered, and evaporated to give an oily residue. The residue was purified via a silica plug using hexanes/ether, 1:2 to give the desired as a white solid product (250 mg, 69 %). ^{1}H NMR (400 MHz, $CDCl_3$) δ 8.96 (d, J = 12.0 Hz, 1H), 8.05 (dd, J = 14.2, 12.0 Hz, 1H), 7.63–7.46 (m, 4H), 5.65 (d, J = 14.2 Hz, 1H), 5.23 (s, 2H), 4.36 (bs, 1H), 4.21–4.07 (m, 1H), 3.65–3.51 (m, 2H), 2.66 (bs, 1H), 1.03 (s, 3H), 0.98 (s, 3H). ^{13}C NMR (101 MHz, $CDCl_3$) δ 171.10, 167.08, 137.27, 137.21, 131.39, 129.08, 124.98, 124.79, 102.06, 78.29, 71.85, 65.16, 39.34, 20.92, 20.17.

Prop-2-en-1-yl-(2*E*)-3-[((2*R*)-2,4-dihydroxy-3,3-dimethylbutanoyl)amino]prop-2-enoate (26d).

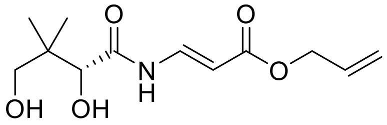

A solution of enamide **25d** (1.6 g, 5.4 mmol) in acetonitrile (100 mL) was treated with $BiCl_3$ (509 mg, 1.6 mmol) and excess water (0.2 mL, 10 mmol). The reaction mixture was stirred at room temperature overnight. Upon disappearance of starting material by thin-layer chromatography analysis, the mixture was filtered through Celite, dried over Na_2SO_4 and filtered once more, then concentrated *in vacuo* by rotary evaporation. Upon purification of the crude residue by column chromatography (silica gel; CH_2Cl_2/EtOAc), the title compound was attained as a light-yellow oil (955.0 mg, 69%). ^{1}H NMR (400 MHz, $CDCl_3$): δ 9.09 (d, *J* = 15.2, 1H), 8.03 (dd, *J* = 14.2, 11.9 Hz, 1H), 6.00–5.88 (m, 1H), 5.64 (d, *J* = 14.2 Hz, 1H), 5.33 (dq, *J* = 17.2, 1.4 Hz, 1H), 5. 24 (dq, *J* = 10.5, 1.3 Hz, 1H), 4.77 (br s, 1H), 4.64 (d, *J* = 5.7 Hz, 2H), 4.18 (d, *J* = 4.3 Hz, 1H), 4.12 (q, *J* = 7.5 Hz, 1H), 3.55 (s, 2H), 3.35 (br s, 1H), 1.02 (s, 3H), 0.98 (s, 3H). MS (ESI+) *m/z* 258.25 $[M+H]^+$.

[(2,2-dimethylpropanoyl)oxy]methyl (2E)-3-[(2S)-4-{[bis({[(2,2-dimethylpropanoyl)oxy]methoxy})-phosphoryl]oxy}-2-hydroxy-3,3-dimethylbutanamido]prop-2-enoate (14a).

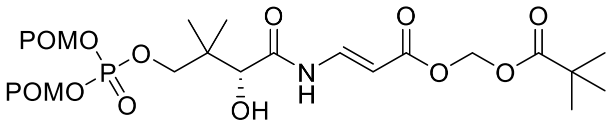

To a solution of **26a** (40 mg, 0.1 mmol) and bis(POM)-phosphoryl chloride (**27**)[3] (200 mg, 0.6 mmol) in ethyl ether (1 mL) was added triethylamine (0.2 mL) slowly. The reaction mixture was allowed to stir at room temperature under a nitrogen atmosphere for two hours. The cloudy reaction was quenched by the addition of aq. NH_4Cl and extracted with EtOAc (3×5 mL). The organic portion was dried over $MgSO_4$, filtered, and evaporated. The residue was purified by preparative TLC twice (with CH_2Cl_2/ether, 3:1 and then ether) to give the desired compound as an oil (30 mg, 39%). ^{1}H NMR (400 MHz, $CDCl_3$) δ 9.17 (d, *J* = 11.9 Hz, 1H), 8.06 (dd, *J* = 14.3, 11.8 Hz, 1H), 5.81 (s, 2H), 5.71–5.54 (m, 4H), 4.28–4.15 (m, 2H), 4.22 (dd, *J* = 10.2, 8.0 Hz, 1H), 4.18 (bs, 1H), 3.65 (dd, *J* = 10.3, 8.7 Hz, 1H), 1.24 (s, 9H), 1.23 (s, 9H), 1.21 (s, 9H), 1.17 (s, 3H), 0.85 (s, 3H). ^{13}C NMR (101 MHz, $CDCl_3$) δ 177.28, 176.97, 176.84, 170.52, 165.88, 138.26, 101.10, 82.99, 79.39, 74.16, 73.31, 40.04, 39.99, 38.77, 26.88, 26.83, 26.80, 21.02, 18.04. HRMS (ESI+) calcd for $C_{27}H_{46}NO_{14}PNa$, 662.2554; found, 662.2545 $[M+Na]^+$.

(5-methyl-2-oxo-2H-1,3-dioxol-4-yl)methyl (2E)-3-[(2S)-4-{[bis({[(2,2-dimethylpropanoyl)oxy]-methoxy})phosphoryl]oxy}-2-hydroxy-3,3-dimethylbutanamido]prop-2-enoate (14b).

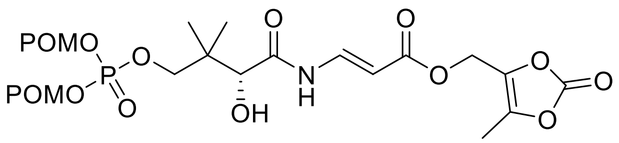

To a suspension of **26b** (40 mg, 0.1 mmol) and bis(POM)-phosphoryl chloride (**27**)[3] (200 mg, 0.6 mmol) in ethyl ether (1 mL) was added triethylamine (0.2 mL) slowly. The reaction mixture was allowed to stir at room temperature under a nitrogen atmosphere for one hour. The reaction was quenched by the addition of aq. NH_4Cl and extracted with EtOAc (3×5 mL). The mixture was dried over $MgSO_4$, filtered, and evaporated. The residue was purified by preparative TLC twice (with ether and then CH_2Cl_2/ether, 3:1) to give the desired compound as a colorless oil (28 mg, 37%). ^{1}H NMR (400 MHz, $CDCl_3$) δ 9.18 (d, *J* = 12.0 Hz, 1H), 8.14–7.98 (m, 1H), 5.73–5.55 (m, 6H), 4.90 (s, 2H), 4.81 (d, *J* = 6.2 Hz, 1H), 3.65 (dd, *J* = 10.3, 8.7 Hz, 1H), 3.24 (bs, 1H), 2.19 (s, 3H), 1.25 (s, 6H), 1.24 (s, 12H), 1.18 (s, 3H), 0.85 (s, 3H). ^{13}C NMR (101 MHz, $CDCl_3$) δ 177.26, 176.86, 170.55, 166.67, 152.27, 138.04, 100.97, 83.08, 83.03, 83.00, 82.95, 82.42, 82.37, 74.20, 74.14, 73.30, 53.40, 40.08, 40.03, 38.77, 26.86, 26.84, 26.82, 21.06, 18.01, 9.39. HRMS (ESI+) calcd for $C_{26}H_{40}NO_{15}PNa$, 660.2033; found 660.2025 $[M+Na]^+$.

[3-(trifluoromethyl)phenyl]methyl (2E)-3-[(2S)-4-{[bis({[(2,2-dimethylpropanoyl)oxy]methoxy})-phosphoryl]oxy}-2-hydroxy-3,3-dimethylbutanamido]prop-2-enoate (14c).

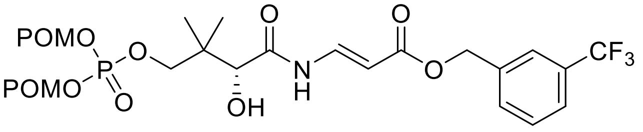

To a suspension of **26c** (60 mg, 0.16 mmol) and bis(POM)-phosphoryl chloride (**27**)[3] (300 mg, 0.9 mmol) in ethyl ether (2 mL) was added triethylamine (0.2 mL) slowly. The reaction mixture was allowed to stir at room temperature under a nitrogen atmosphere overnight. The reaction was quenched by the addition of aq. NH_4Cl and extracted with EtOAc (3×5 mL). The mixture was dried over $MgSO_4$, filtered, and evaporated. The residue was purified by normal phase column chromatography (using ether) followed by preparative TLC (using CH_2Cl_2/ether, 4:1) to give the desired compound as an oil (25 mg, 24%). ^{1}H NMR (400 MHz, $CDCl_3$) δ 9.13 (d, *J* = 11.9 Hz, 1H), 8.05 (dd, *J* = 14.2, 11.9 Hz, 1H), 7.62 (s, 1H), 7.60–7.45 (m, 3H), 5.73–5.58 (m, 5H), 5.23 (s, 2H), 4.75 (bs, 1H), 4.28–4.16 (m, 2H), 3.69–3.58 (m, 1H), 1.25 (s, 9H), 1.23 (s, 9H), 1.19 (s, 3H), 0.85 (s, 3H). ^{13}C NMR (101 MHz, $CDCl_3$) δ 177.00, 176.86, 170.47, 166.98, 137.34, 137.32, 131.40, 129.05, 124.98, 124.95, 124.91, 124.87, 124.82, 124.78, 124.74, 101.89, 83.08, 83.03, 83.00, 82.95, 74.24, 74.19, 73.27, 65.05, 40.08, 40.04, 38.80, 26.84, 26.81, 21.06, 17.98. HRMS (ESI) calcd for $C_{29}H_{41}F_3NO_{12}P$, 684.2397; found, 684.2397 $[M+H]^+$.

Prop-2-en-1-yl (2E)-3-[(2R)-4-{[bis({[(2,2-dimethylpropanoyl)oxy]methoxy})phosphoryl]oxy}-2-hydroxy-3,3-dimethylbutanamido]prop-2-enoate (14d).

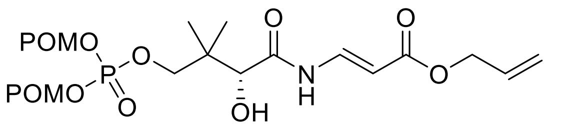

Bis(POM) phosphate (**28**)[3] (798 mg, 2.45 mmol) was added to a stirred solution of HATU (1.4 g, 3.7 mmol) and DIPEA (1.3 mL, 7.3 mmol) in dry DMF (10 mL). The solution was stirred at room temperature for 10 minutes, followed by the addition of 1,3-diol **26d** (630 mg, 2.4 mmol). The reaction was stirred at room temperature overnight, with product formation monitored by thin-layer chromatography and LC-MS. Upon completion of the reaction, the solvent was removed *in vacuo* by rotary evaporation, followed by purification using column chromatography (silica gel; hexanes/EtOAc) to give phosphorylated product **14d** as a colorless oil (124 mg, 9%). ^{1}H NMR (400 MHz, $CDCl_3$): δ 9.09 (s, 1H), 8.04 (d, *J* = 15.1 Hz, 1H), 5.94 (d, *J* = 8.0 Hz, 2H), 5.64 (d, *J* = 8.0 Hz, 2H), 5.34 (m, *J* = 16.8 Hz, 1H), 5.23 (d, *J* = 15.1 Hz, 1H), 5.31 (s, 1H), 5.29 (s, 1H), 4.77 (s, 1H), 4.62 (s, 1H), 4.21 (s, 1H), 3.75 (bs, 1H), 1.83 (s, 6H), 1.19 (s, 18H). MS (ESI+) *m/z* 566.25 $[M+H]^+$.

(*E*)-3-{(*R*)-4-[Di(2,2-dimethylvaleroxy)methoxyphosphoryloxy]-2-hydroxy-3,3-dimethylbutyryl-amino}acrylic acid (14e).

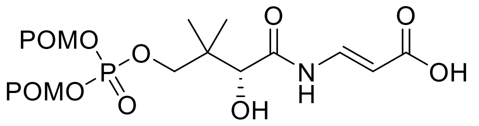

To a solution of allyl ester **14d** (110 mg, 0.19 mmol) in THF (1.5 mL) was added *N*-hydroxyphthalimide (95 mg, 0.58 mmol). After this stirred for 5 minutes, $Pd(PPh_3)_4$ (45 mg, 0.04 mmol) was added. The reaction was stirred in the dark at room temperature overnight, then filtered and concentrated *in vacuo* by rotary evaporation. The crude product was purified by column chromatography (silica gel; EtOAc) to afford target compound **14e** as a yellow oil (21 mg, 21%). ^{1}H NMR (400 MHz, $CDCl_3$): δ 7.69 (d, *J* = 15.1 Hz, 1H), 7.48 (d, *J* = 8.0 Hz, 2H), 7.44 (d, *J* = 8.0 Hz, 2H), 5.66 (d, *J* = 15.1 Hz, 1H), 4.02 (s, 1H), 3.59 (s, 1H), 1.43 (s, 6H), 1.20 (s, 18H). ^{13}C NMR (400 MHz, $CDCl_3$): δ 176.82, 170.41, 163.20, 132.92, 103.70, 93.70, 83.03, 82.95, 38.65, 26.81, 18.17. MS (ESI+) *m/z* 548.20 $[M+Na]^+$.

Overexpression and purification of enzymes. All expressed proteins contained *N*-terminal 6×histidine tags for purification by immobilized metal affinity chromatography (IMAC). *Sa*PanK_{II} and *Sa*CoaBC were expressed and purified as previously described.[7]

An expression plasmid for *Mt*PanK_I was prepared by GenScript; the Mtb *coaA* gene sequence was codon optimized for expression in *E. coli*, and then synthesized with NdeI (CATATG, the underlined ATG being the gene's start codon) and XhoI (CTCGAG) restriction sites on the 5′ and 3′-

termini respectively. The gene's stop codon was retained. The synthesized gene was then inserted into the NdeI and XhoI sites of the pET28a(+) vector, to give the pET28a-*MtbcoaA* expression plasmid. *Mt*PanK_I was expressed by transforming the plasmid into competent *E. coli* BL21 (DE3), which was grown in 2×YT media (16 g tryptone, 10 g yeast extract, 5 g NaCl per litre) at 37°C until induction with 0.5 mM IPTG at OD_{600} = 0.6. After expression at 16°C overnight, 250 mL culture media were centrifuged and pelleted and the collected pellet used immediately or stored at -80°C until purification.

*Mt*CoaBC was expressed by transforming pET-28a_*MtcoaBC*[8] (a kind gift from Tom Blundell, University of Cambridge) into competent *E. coli* BL21 (DE3) for the heterologous expression. Transformed cells were grown in Luria-Bertani (LB) media at 37°C, and expression was induced with 1 mM IPTG at OD_{600} = 0.6. After expression at 20°C overnight, 500 mL cell culture was centrifuged and the collected pellet used immediately or stored at -80°C until purification.

For protein purification, cell pellets were resuspended in sonication buffer (20 mM Tris-HCl pH 7.9, 500 mM NaCl, 5 mM imidazole, 5% v/v glycerol) at 10 mL/1 g cell paste, followed by sonication to completely lyse the cells. The cell lysate was centrifuged to pellet the cell debris and the supernatant, containing the soluble protein fraction, was loaded onto a 1 mL HiTrap Chelating HP column (Cytiva Life Sciences) on an ÄKTA start protein purification system. Non-specific proteins were washed off the column with sonication buffer that contained 75 mM imidazole. The protein of interest was eluted from the column using elution buffer (sonication buffer containing 500 mM imidazole). All buffers contained 5% v/v glycerol, except the *Mt*PanK_I elution buffer that contained 10% v/v glycerol. The sproteins were then subjected to buffer exchange on a 5 mL HiTrap Desalting column (Cytiva Life Sciences) into their final storage buffer: for *Mt*PanK_I, the buffer was stored in 50 mM Tris-HCl pH 7.5, 300 mM NaCl, 0.1 mM EDTA, 10% v/v glycerol; for *Mt*CoaBC the buffer was 25 mM Tris-HCl pH 8.0, 5 mM $MgCl_2$, 150 mM NaCl, 0.5 mM TCEP, 5% v/v glycerol. Before final storage, *Mt*CoaBC was incubated with flavin mononucleotide (FMN) in a 10× molar excess for two hours on ice. Excess FMN was removed via buffer exchange on a 5 mL HiTrap Desalting column. Protein samples were aliquoted and subjected to no more than a single freeze thaw cycle. All protein purities were confirmed via SDS-PAGE analysis. Protein concentrations were determined with Bradford reagent and a bovine serum albumin (Fraction V) standard curve was used as reference.

PPCS activity assay. PPCS activity was continuously monitored at 340 nm by coupling the production of pyrophosphate to the oxidation of NADH using the pyrophosphate reagent (Sigma-Aldrich, cat. #P7275). Reactions were initiated by addition of 120 μL of a master assay mix that was pre-incubated at 37°C for 15 min to the wells of a clear, flat bottom 96-well plate (Greiner Bio-One) containing 30 μL

P-Pan (**5**) (concentrations ranging between 12.5 µM to 500 µM), the final assay volume being 150 µL. The assay components and their final concentrations were: 50 mM Tris–HCl buffer (pH 7.6), 2.5 mM $MgCl_2$, 20 mM KCl, 1.0 mM CTP, 1.0 mM L-Cys, 2.5 mM DTT and CoaBC enzyme (25.5 nM *Sa*CoaBC or 38.3 nM *Mt*CoaBC); each 150 µL contained 60 µL pyrophosphate reagent. The rate of NADH oxidation was monitored in triplicate for each substrate concentration by measuring the absorbance at 340 nm each 10 seconds for 15 minutes using a Varioskan Flash spectrophotometer (Thermo Fisher Scientific). Initial velocities were calculated (a molar extinction coefficient of 6220 $M^{-1}.cm^{-1}$ was used for NADH) and plotted against each P-Pan concentration, and the data fitted to the Michaelis-Menten equation (Eqn. S1) using SigmaPlot 14.0.

$$v = \frac{V_{max} \times [S]}{K_m + [S]} \qquad \text{Eqn.S1}$$

PPCS inhibition assay and data analyses. PPCS inhibition assays were performed as previously described,[7] using the same method as for determining PPCS activity described above, except that the wells of the 96-well plate contained 30 µL of a mixture of P-Pan (**5**) (250 µM) and P-CJ (**10**) (in concentrations ranging between 0.025 µM to 1.6 µM). From the resulting progress curves the activity at each inhibitor concentration was calculated by linear regression. The plot of the fractional activity (inhibited rate as a fraction of the uninhibited rate) at each inhibitor concentration was used to calculate the value of K_i^{app} by fitting the data to the Morrison equation for tight-binding inhibitors (Eqn. S2) using SigmaPlot 14.0. The K_i^{app} was then converted to K_i using the Cheng-Prusoff equation appropriate for competitive inhibitors (Eqn. S3) and the appropriate K_M value.

$$\frac{v_i}{v_0} = 1 - \frac{([E]+[I]+K_i^{app}) - \sqrt{([E]+[I]+K_i^{app})^2 - 4[E][I]}}{2[E]} \qquad \text{Eqn. S2}$$

$$K_i^{app} = K_i\left(1 + \frac{[S]}{K_m}\right) \qquad \text{Eqn. S3}$$

Protein temperature melt curve determinations using circular dichroism. Initial protein melting curves were determined by measuring the change in circular dichroism at 220 nm as the temperature of protein samples were increased from 35°C to 80°C at a rate of 1°C per minute. All measurements were made in an Applied Photophysics Chirascan-Plus CD Spectrometer using a 0.5 mm cuvette. Four separate samples of each protein were prepared, all containing 10 µM CoaBC and 1.0 mM $MgCl_2$ in 50 mM Tris–HCl buffer (pH 7.6). The first sample contained no additional components, while the other three contained 150 µM CTP, 150 µM each of P-Pan (**5**) and CTP, or 150 µM each of P-CJ (**10**) and CTP, respectively. The final volume of all samples was 240 µL. A sampling time of 0.5 seconds per

point was employed. A temperature probe was used in the sample solutions and the preset instrument temperatures were replaced with temperatures measured each minute by the probe for higher accuracy. The T_m for each sample was subsequently determined by normalizing the melting curve data, and fitting the resulting curves simultaneously to Equations 4, 5 and 6 (the Gibbs-Helmholtz equation) using SigmaPlot 14.0, where *lb* and *rb* are the equations for the best linear fits to the left and right baselines of the curves, respectively. The following parameter constraints were used for the curve fit: Van't Hoff enthalpy (ΔH_{vH}) > 0; T_m > 0 and ΔC_p > 0. For *Sa*CoaBC, accurate T_m determinations were not possible since the protein was not fully denatured at the highest temperature that can be obtained in the spectrometer. The T_m-values were therefore estimated by normalizing the melting curve data and determining the first derivatives of the curves.

$$y = FF \times (lb - rb) + rb \qquad \text{Eqn. S4}$$

$$FF = \frac{1}{1+\left(\frac{1}{e^{\left(\frac{\Delta G}{1.9872\times T}\right)}}\right)} \qquad \text{Eqn. S5}$$

$$\Delta G = \Delta H_{\vartheta H}\left(1 - \frac{T}{T_m}\right) - \Delta C_p\left(T_m - T ln\frac{T}{T_m}\right) \qquad \text{Eqn. S6}$$

PanK activity assays. PanK activity was characterised by the continuous, enzyme coupled pyruvate kinase (PK)/lactate dehydrogenase (LDH) kinase assay as previously described.[9] Each 300 µL reaction contained 50 mM Tris-HCl pH 7.6, 10 mM $MgCl_2$, 20 mM KCl, 0.5 mM NADH, 2 mM PEP, 0.01 U/µL PK and 0.01 U/µL LDH, 0.01 mg/mL of either *Sa*PanK$_{II}$ or *Mt*PanK$_{I}$ and ATP (1.5 mM ATP for *Sa*PanK$_{II}$ assays, and 3.0 mM for assays with *Mt*PanK$_{I}$). Reactions were initiated by the addition of 400 µM of putative substrate, these being one of the Pan-CMP bisubstrate mimics **12**, or 400 µM of Pan (**2**). Reactions were monitored at 25°C by measuring the absorbance at 340 nm each 6 seconds for 5 minutes using a Varioskan Flash spectrophotometer (Thermo Fisher Scientific). Each reaction was performed in triplicate. The initial rate of activity at each substrate concentration was obtained by linear regression of the plots in SigmaPlot 14.0; the activity was reported as a percentage of the activity with Pan.

Systems-based PPCS inhibition assay. For the systems-based inhibition assay we chose conditions that closely reflect those expected to be observed during normal growth. Specifically, the ratio of PanK and CoaBC was based on the protein abundance database, PaxDb (4.0) a comprehensive integrated dataset of protein abundance based on the weighted averages of the data of numerous proteomics studies; it represents the best available estimate of the ratios of the members of an organism's proteome.[10] For *S. aureus* and Mtb the relative integrated abundances of PanK and CoaBC are 1:9 and 1:2.2 respectively;

we therefore used these ratios for reconstitution of the truncated pathways. For the *S. aureus* pathway assays, the reaction mix contained 50 mM Tris–HCl (pH 7.6), 10 mM $MgCl_2$, 20 mM KCl, 2 mM TCEP, 1 mM L-cysteine, 1 mM CTP, 1.5 mM ATP, 1 mM phosphoenolpyruvate (PEP), 0.04 U/µL PK, 0.01 mg/mL *Sa*PanK$_{II}$ and 0.09 mg/mL *Sa*CoaBC, and 400 µM of one of the bisubstrate mimics **12**. The reaction mix was incubated at 37°C for 15 minutes prior to the initiation with 500 µM Pan (**2**). Reactions were then monitored over time at 37 °C; for each time point, a separate 50 µL reaction mix was prepared.

For the Mtb pathway assays, reactions mixtures were the same as for the *S. aureus* assay, except PEP and PK were omitted as this caused a decrease in P-PanSH production (data not shown), therefore 3 mM ATP was added to keep ATP concentration in excess. FMN was also added at a 10 times molar excess of *Mt*CoaBC. Reaction mixtures were incubated at 25°C (the experimentally determined optimal temperature for this system) for 15 minutes before initiating the reactions with 500 µM Pan (**2**). Reactions were then monitored over time at 25°C; for each time point, a separate 50 µL reaction mix was prepared.

Reactions were stopped at the appropriate time points by acid precipitation with the addition of 10 µL 90 % (w/v) trichloroacetic acid (TCA). Next the samples were treated and prepared for HPLC analysis as previously described.[11] Briefly, each acid-precipitated reaction was neutralised by addition of 40 µL 2.25 M NH_4OAc. The precipitated proteins were removed by centrifugation at 16000×g for 10 minutes, after which 7 µL of the supernatant was removed and added to a new tube before derivatization with CPM, a thiol probe that fluoresces upon reaction with thiolated metabolites. Each derivatization reaction contained 7 µL supernatant, 21 µL acetonitrile (30% v/v final), 41.5 µL H_2O and 1.5 µL 10 mM CPM (206 µM final) in a final volume of 70 µL and was left overnight at room temperature. All samples were analysed by HPLC and quantified as previously described.[11]

DSF binding assays. The DSF optimization and screening was set up based on literature protocols.[12,13] First, the enzyme and dye concentrations were optimized in a 96-well low profile, non-skirted, clear PCR plate (NEST) and the combination was chosen that gave the highest fluorescence as measured on a StepOnePlus Real-Time PCR System (Applied Biosystems). The final concentrations of the various components were: 10 µM enzyme, 100 µM inhibitor or substrate, 5× SYPRO Orange dye, 25 mM Tris–HCl, 5 mM $MgCl_2$, 150 mM NaCl, 5% v/v glycerol and 5% v/v DMSO. Assays were conducted by adding all buffer components and enzyme to each well, followed by addition of the SYPRO Orange dye, and finally the inhibitor or substrate. The final reaction volume was 20 µL. The 96-well plate was then sealed with PCR sealing film and incubated at room temperature for 10 minutes before running a thermal melt protocol on a CFX Opus 96 Real-Time PCR System (Bio-Rad). The fluorescence was

monitored on all channels with temperature being ramped from 25°C to 95°C at 0.2°C per cycle. The CFX Maestro software package was used to plot the generated curves and their first derivatives from which the T_m could be extracted. This is automatically performed when the data is imported to the software. Dye–inhibitor interaction controls were also included for each run and compound. All melting points were determined in triplicate.

***S. aureus* whole cell inhibition.** Minimum inhibitory concentrations (MICs) towards *S. aureus* was determined using the broth microdilution method according to guidelines of the Clinical and Laboratory Standards Institute (CLSI).[14] Pan-CMP bisubstrate analogues **12** were evaluated against *S. aureus* RN4220 grown in minimal media prepared as described by Rudin et al.[15] Masked P-CJ prodrugs **14** were evaluated against *S. aureus* Xen29, a bioluminescent derivative of methicillin-susceptible *S. aureus* strain ATCC 12600), grown in tryptic soy broth (TSB). The indicator strain was grown overnight (18 h) at 37°C under aerobic conditions. The medium was then inoculated (5 $\log_{10}$ CFU/mL) and incubated under static conditions at 37°C for 24 h in the presence of the various compounds at the desired concentration. After incubation (24 h), growth of indicator strains was assessed spectrophotometrically at 600 nm and compared to the negative (uninoculated media) and positive controls (no inhibitors). Data was normalised to the positive control to obtain percentage growth. The MICs were defined as the lowest concentration that inhibited visible growth. Representative dose-response curves for CJ-15,801 (**4**), **14a** and **14b** were prepared by fitting the data to a sigmoidal model with the limits set to 100 and 0, respectively. Due to log transformation the 0 data point was transformed to next magnitude i.e., 0.1. All tests on Pan-CMP bisubstrate analogues **12** were performed at least in duplicate; all tests on other compounds were done in triplicate.

Mtb whole cell inhibition. The *coaBC* Tet-OFF hypomorph used in this study was constructed as previously described,[16] and derived from the virulent, PDIM-producing parental strain, Mtb H37RvMA.[17] All strains were routinely grown in Difco Middlebrook 7H9 broth (BD) supplemented with 10% Middlebrook albumin-dextrose-catalase (ADC) enrichment (BD), 0.2% glycerol (Sigma-Aldrich), and 0.05% Tween-80. Where alternative media were used, strains were first grown to mid-log phase in 7H9 broth supplemented with 10% ADC, 0.2% glycerol and 0.05% Tween-80 then washed twice with, and resuspended in, the specified media. For media containing acetate as the sole carbon source, Difco Middlebrook 7H9 broth was supplemented with 0.5% BSA, 0.08% NaCl, 0.05% tyloxapol, and 10 mM acetate. GAST/Fe was prepared by combining 0.03% Bacto Casitone (Difco), 0.005% ferric ammonium citrate, 0.4% dibasic potassium phosphate, 0.2% citric acid, 0.1% L-alanine,

0.12% magnesium chloride hexahydrate, 0.06% potassium sulphate, 0.2% ammonium chloride, 0.018% of a 1% sodium hydroxide solution, 1% glycerol, and 0.05% Tween-80. Hygromycin and kanamycin were used at concentrations of 50 and 25 μg/mL respectively. In order to repress the expression of target genes in cells expressing revTetR, cultures were grown in the absence of the ATc inducer to OD600 ≈ 0.2 prior to dilution in the specified media containing ATc at concentrations up to 200 ng/mL in order to transcriptionally silence *coaBC*. To avoid inactivation of the inducer, all cultures containing ATc were incubated in the dark, and exposure of the cultures to light was minimized.

REFERENCES

(1) Domingo, R.; van der Westhuyzen, R.; Hamann, A. R.; Mostert, K. J.; Barnard, L.; Paquet, T.; Tjhin, E. T.; Saliba, K. J.; van Otterlo, W. A. L.; Strauss, E. Overcoming Synthetic Challenges in Targeting Coenzyme A Biosynthesis with the Antimicrobial Natural Product CJ-15,801. *Med. Chem. Commun.* **2019**, *10* (12), 2118–2125. https://doi.org/10.1039/C9MD00312F.

(2) Hata, T.; Yamamoto, I.; Sekine, M. A New Method for the Synthesis of 5′-Amino-Nucleosides and Their Phosphoramidate Derivatives. *Chem. Lett.* **1976**, *5* (6), 601–604. https://doi.org/10.1246/cl.1976.601.

(3) Hwang, Y.; Cole, P. A. Efficient Synthesis of Phosphorylated Prodrugs with Bis(POM)-Phosphoryl Chloride. *Org. Lett.* **2004**, *6* (10), 1555–1556. https://doi.org/10.1021/ol049714v.

(4) Meier, J. L.; Mercer, A. C.; Rivera, H.; Burkart, M. D. Synthesis and Evaluation of Bioorthogonal Pantetheine Analogues for in Vivo Protein Modification. *J. Am. Chem. Soc.* **2006**, *128* (37), 12174–12184. https://doi.org/10.1021/ja063217n.

(5) Weir, J. R.; Patel, B. A.; Heck, R. F. Palladium-Catalyzed Triethylammonium Formate Reductions. 4. Reduction of Acetylenes to Cis-Monoenes and Hydrogenolysis of Tertiary Allylic Amines. *J. Org. Chem.* **1980**, *45* (24), 4926–4931. https://doi.org/10.1021/jo01312a021.

(6) Aquino, F. P., Horst; Walther, Willy; Plattner, Dietmar A; Bonrath, Werner. A Convenient Dehydration Procedure for the Synthesis of Enantiomerically Pure Cyanohydrins. *Synthesis* **2000**, *2000* (05), 731–737. https://doi.org/10.1055/s-2000-6403.

(7) van der Westhuyzen, R.; Hammons, J. C.; Meier, J. L.; Dahesh, S.; Moolman, W. J. A.; Pelly, S. C.; Nizet, V.; Burkart, M. D.; Strauss, E. The Antibiotic CJ-15,801 Is an Antimetabolite That Hijacks and Then Inhibits CoA Biosynthesis. *Chem. Biol.* **2012**, *19* (5), 559–571. https://doi.org/10.1016/j.chembiol.2012.03.013.

(8) Mendes, V.; Green, S. R.; Evans, J. C.; Hess, J.; Blaszczyk, M.; Spry, C.; Bryant, O.; Cory-Wright, J.; Chan, D. S.-H.; Torres, P. H. M.; Wang, Z.; Nahiyaan, N.; O'Neill, S.; Damerow, S.; Post, J.; Bayliss, T.; Lynch, S. L.; Coyne, A. G.; Ray, P. C.; Abell, C.; Rhee, K. Y.; Boshoff, H. I. M.; Barry, C. E.; Mizrahi, V.; Wyatt, P. G.; Blundell, T. L. Inhibiting *Mycobacterium tuberculosis* CoaBC by Targeting an Allosteric Site. *Nat Commun* **2021**, *12* (1), 143. https://doi.org/10.1038/s41467-020-20224-x.

(9) Brand, L. A.; Strauss, E. Characterization of a New Pantothenate Kinase Isoform from *Helicobacter pylori*. *J. Biol. Chem.* **2005**, *280* (21), 20185–20188. https://doi.org/10.1074/jbc.C500044200.

(10) Wang, M.; Herrmann, C. J.; Simonovic, M.; Szklarczyk, D.; Von Mering, C. Version 4.0 of PaxDb: Protein Abundance Data, Integrated across Model Organisms, Tissues, and Cell-lines. *Proteomics* **2015**, *15* (18), 3163–3168. https://doi.org/10.1002/pmic.201400441.

(11) Goosen, R.; Strauss, E. Simultaneous Quantification of Coenzyme A and Its Salvage Pathway Intermediates in *In Vitro* and Whole Cell-Sourced Samples. *RSC Adv.* **2017**, *7* (32), 19717–19724. https://doi.org/10.1039/C7RA00192D.

(12) Niesen, F. H.; Berglund, H.; Vedadi, M. The Use of Differential Scanning Fluorimetry to Detect Ligand Interactions That Promote Protein Stability. *Nat Protoc* **2007**, *2* (9), 2212–2221. https://doi.org/10.1038/nprot.2007.321.

(13) Mashalidis, E. H.; Śledź, P.; Lang, S.; Abell, C. A Three-Stage Biophysical Screening Cascade for Fragment-Based Drug Discovery. *Nat Protoc* **2013**, *8* (11), 2309–2324. https://doi.org/10.1038/nprot.2013.130.

(14) CSLI. Performance Standards for Antimicrobial Susceptibility Testing. 30th Ed. CSLI Supplement M100. 2020, Wayne, PA: Clinical and Laboratory Standards Institute.

(15) Rudin, L.; Sjöström, J. E.; Lindberg, M.; Philipson, L. Factors Affecting Competence for Transformation in *Staphylococcus aureus*. *J Bacteriol* **1974**, *118* (1), 155–164. https://doi.org/10.1128/jb.118.1.155-164.1974.

(16) Evans, J. C.; Trujillo, C.; Wang, Z.; Eoh, H.; Ehrt, S.; Schnappinger, D.; Boshoff, H. I. M.; Rhee, K. Y.; Barry, C. E.; Mizrahi, V. Validation of CoaBC as a Bactericidal Target in the Coenzyme A Pathway of *Mycobacterium tuberculosis*. *ACS Infect. Dis.* **2016**, *2* (12), 958–968. https://doi.org/10.1021/acsinfecdis.6b00150.

(17) Ioerger, T. R.; Feng, Y.; Ganesula, K.; Chen, X.; Dobos, K. M.; Fortune, S.; Jacobs, W. R.; Mizrahi, V.; Parish, T.; Rubin, E.; Sassetti, C.; Sacchettini, J. C. Variation among Genome Sequences of H37Rv Strains of *Mycobacterium tuberculosis* from Multiple Laboratories. *Journal of Bacteriology* **2010**, *192* (14), 3645–3653. https://doi.org/10.1128/jb.00166-10.
